# Characterization and structural analysis of the *endo*-1,4-*β*-xylanase GH11 from the hemicellulose-degrading *Thermoanaerobacterium saccharolyticum* useful for lignocellulose saccharification

**DOI:** 10.1038/s41598-023-44495-8

**Published:** 2023-10-13

**Authors:** In Jung Kim, Soo Rin Kim, Kyoung Heon Kim, Uwe T. Bornscheuer, Ki Hyun Nam

**Affiliations:** 1 Department of Food Science and Technology, Institute of Agriculture and Life Science, Gyeongsang National University, Jinju, 52828 South Korea; 2 Department of Biotechnology and Enzyme Catalysis, Institute of Biochemistry, University of Greifswald, Felix-Hausdorff-Str. 4, 17489 Greifswald, Germany; 3 School of Food Science and Biotechnology, Kyungpook National University, Daegu, 41566 South Korea; 4 Department of Biotechnology, Graduate School, Korea University, Seoul, 02841 South Korea; 5 College of General Education, Kookmin University, Seoul, 02707 South Korea

**Keywords:** Biochemistry, Structural biology

## Abstract

Xylanases are important for the enzymatic breakdown of lignocellulose-based biomass to produce biofuels and other value-added products. We report functional and structural analyses of TsaGH11, an *endo*-1,4-*β*-xylanase from the hemicellulose-degrading bacterium, *Thermoanaerobacterium saccharolyticum*. TsaGH11 was shown to be a thermophilic enzyme that favors acidic conditions with maximum activity at pH 5.0 and 70 °C. It decomposes xylans from beechwood and oat spelts to xylose-containing oligosaccharides with specific activities of 5622.0 and 3959.3 U mg^−1^, respectively. The kinetic parameters, *K*_*m*_ and* k*_*cat*_ towards beechwood xylan, are 12.9 mg mL^−1^ and 34,015.3 s^−1^, respectively, resulting in *k*_*cat*_*/K*_*m*_ value of 2658.7 mL mg^−1^ s^−1^, higher by 10^2^–10^3^ orders of magnitude compared to other reported GH11s investigated with the same substrate, demonstrating its superior catalytic performance. Crystal structures of TsaGH11 revealed a β-jelly roll fold, exhibiting open and close conformations of the substrate-binding site by distinct conformational flexibility to the thumb region of TsaGH11. In the room-temperature structure of TsaGH11 determined by serial synchrotron crystallography, the electron density map of the thumb domain of the TsaGH11 molecule, which does not affect crystal packing, is disordered, indicating that the thumb domain of TsaGH11 has high structural flexibility at room temperature, with the water molecules in the substrate-binding cleft being more disordered than those in the cryogenic structure. These results expand our knowledge of GH11 structural flexibility at room temperature and pave the way for its application in industrial biomass degradation.

## Introduction

As the trend of global industrialization continues, there is a constant increase in demand for energy, requiring more fuel production^1^. Traditional energy sources such as fossil fuels, which are non-renewable and unsustainable, negatively influence regional and global environments through air pollution, climate change, and energy conflicts^2,3^. To overcome these environmental issues and simultaneously cater to the increasing demand for energy, global efforts are being made to reduce the consumption of fossil fuels by switching to renewable and eco-friendly energy sources. Sustainable energy from biomass and biomass waste, solar, hydro, wind, geothermal, and ocean sources are expected to play a significant role in the future as alternative energy sources. Among these, lignocellulosic biomass feedstock, readily available in nature in the form of agricultural and wood wastes, for example, is a promising alternative to replace fossil fuels as a primary source of carbohydrates. Lignocellulose-based biomass can meet global sustainability standards and fulfill the raw material demand of the processing industry while supporting the transition from a linear to a circular economy^4^.

Lignocellulose is a major component of plant cell walls and consists mainly of cellulose (40–50%), hemicellulose (25–30%), lignin (15–20%), minor portions of pectins, waxes, and mineral salts^5,6^. A complex and heterogeneous three-dimensional network is formed via covalent and non-covalent interactions between each of these components^7–9^. Xylan, the main component of hemicellulose, is the second most abundant polysaccharide on Earth after cellulose, accounting for approximately one-third of the Earth's renewable organic carbon sources^8,10^. Xylan is a complex heteropolysaccharide composed of monosaccharides (i.e., l-arabinose, d-galactose, and d-mannose) and organic acids (i.e., acetic acid, ferulic acid, and glucuronic acid) that bind to glycosides through esterification^11^. More specifically, xylan consists of a linear backbone made up of d-xylose with β-1,4-linkage, which is often branched with other sugars such as α-arabinose and/or organic acids. Highly varying xylan structures—depending on vegetal origin and chemical pretreatment methods—as well as its heterogeneous nature, cause limited degradation. It is thus necessary to develop an efficient saccharification process for xylan to obtain useful sugars applicable in various industrial sectors, including biofuels, food/feed, and pulp^9,12^.

Xylanases are glycoside hydrolases (GHs) that can cleave the β-1,4-glycoside linkages in the xylan backbone. They are synthesized by various organisms and exhibit diverse substrate specificities, catalytic mechanisms, and physicochemical properties. Based on the Carbohydrate-Active enZYme (CAZy) database, GH families 5, 7, 8, 10, 11, and 43 are classified as xylanases, of which the endo-type GH11 family is considered a “true xylanase” that specifically recognizes xylan substrates but not cellulose^13^. Xylanases have attracted increasing attention due to their potential for use in various applications. Consequently, the characteristics and structures of xylanase derived from various organisms have been continuously analyzed, with efforts being made to discover new xylanases with novel and superior characteristics^14^. Functional and structural studies of xylanases are thus important, particularly for developing efficient processes of enzyme-based lignocellulosic biomass degradation for sustainable economy^8,15^.

Thermophilic lignocellulose-degrading bacteria have the natural ability to digest and ferment polysaccharides (i.e., cellulose and hemicellulose) at elevated temperatures and are considered an important source of robust CAZymes^16,17^. *Thermoanaerobacterium saccharolyticum* is a thermophilic anaerobic bacterium that grows in a temperature range of 45–70 °C and can ferment various carbohydrates such as starch, xylan, glucose, cellobiose, xylose, arabinose, mannose, and galactose; whereas it cannot degrade crystalline cellulose^18^. In the genome of *T. saccharolyticum*, three genes (*Tsac_0897*, *Tsac_1459*, and *Tsac_1460*) are annotated as putative endo-xylanases in the UniProt protein database of which *Tsac_1459* and *Tsac_1460* belong to GH10, while *Tsac_0897* belongs to GH11. The molecular properties of the *endo*-1,4-β-xylanase from *T. saccharolyticum,* belonging to the GH11 family has not been characterized yet.

In this study, we report the functional and structural analysis of the GH11 endo-xylanase from the thermophilic and hemicellulolytic *T. saccharolyticum* (TsaGH11). Here, we not only present beneficial aspects of TsaGH11 for industrial applications, but also provide structural insights into its molecular properties. We determined the crystal structure of TsaGH11 at cryogenic and room temperatures, and investigated the flexibility of the substrate binding site pocket of TsaGH11. Our results expand our knowledge of the structure–function relationship of the xylanases and provide a template for protein engineering aimed at hemicellulosic sugar production for sustainable bioprocesses in the future.

## Results

### Functional characterization of TsaGH11

**Figure 1:**
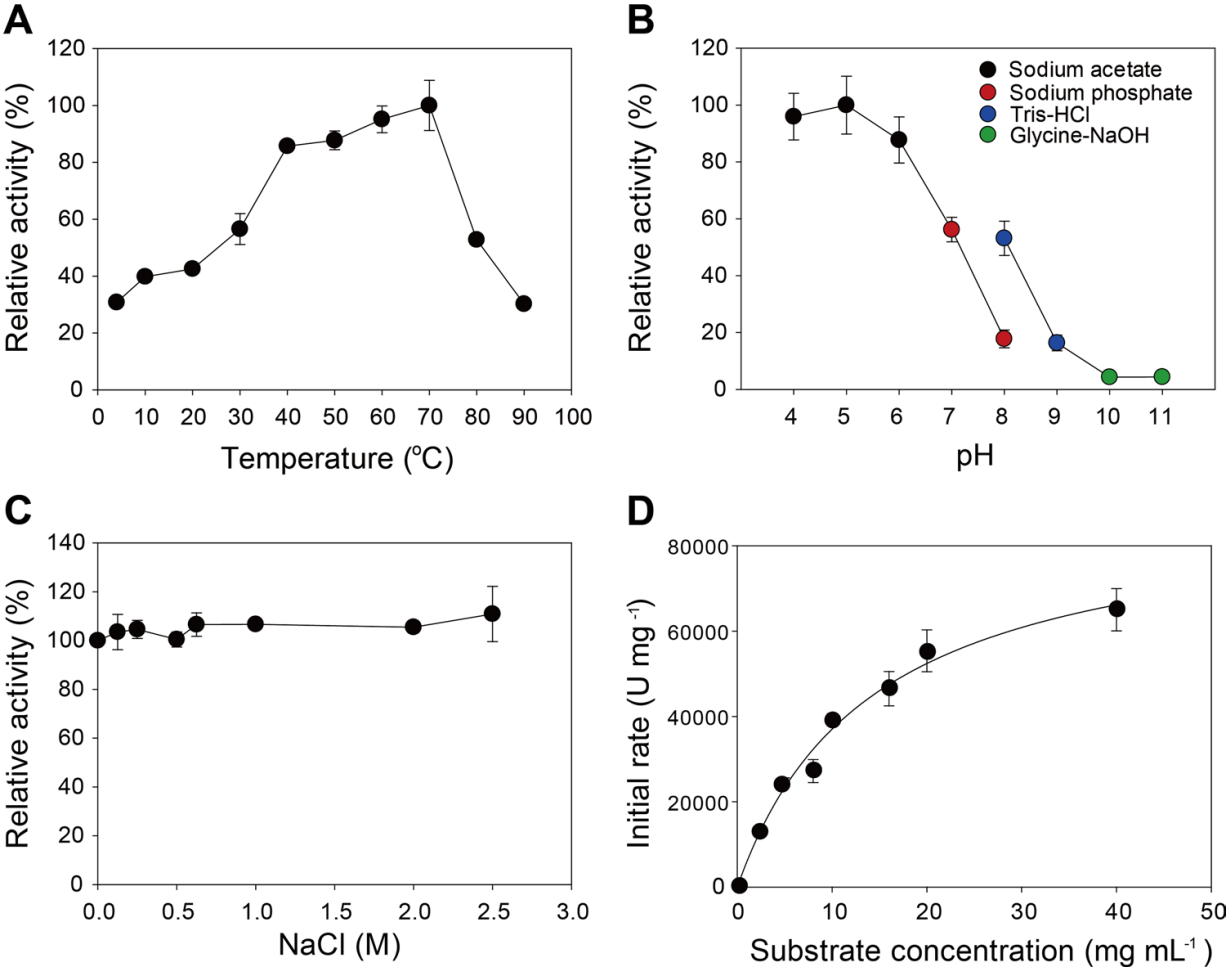
Characterization of the functional properties of TsaGH11. (**A**) The optimal temperature for TsaGH11 hydrolysis of beechwood xylan was examined in the range of 4 to 90 °C in 50 mM sodium acetate buffer (pH 5.0). (**B**) The optimal pH for TsaGH11 hydrolysis of beechwood xylan was assessed at 70 °C at different pHs using 50 mM sodium acetate (pH 4.0–5.0), 50 mM sodium phosphate (pH 6.0–8.0), 50 mM Tris–HCl (pH 8.0–9.0), and 50 mM glycine–NaOH (pH 10.0–11.0). (**C**) The effect of salt on the TsaGH11 activity on beechwood xylan was determined at different concentrations of NaCl (0–2.5 M) at 70 °C and pH 5.0. (D) Enzyme kinetics. Enzyme reactions were performed with various concentrations (0 to 27 mg mL^−1^) of beechwood xylan under optimal conditions (pH 5.0 and 70 °C). The relative activity (%) in panels (A and B) is designated as the activity relative to that determined at the optimal temperature (70 °C) and pH-values (pH 5.0), where the specific activity was 5622.0 U mg^−1^. The relative activity (%) in panel (**C**) is designated as the activity relative to that determined without the supplementation of NaCl with a specific activity of 5622.0 U mg^−1^. The data indicate the means ± standard deviations of three replicates.

When the enzyme activity toward xylans from beechwood and oat spelts was evaluated, TsaGH11 displayed a higher activity towards beechwood xylans than for oat spelts, with specific activities of 5622.0 ± 290.2 and 3959.3 ± 745.0 U mg^−1^, respectively. This indicates that glucuronoxylan is a more favorable substrate for TsaGH11 compared to arabinoxylan. Next, the effects of temperature and pH on the enzymatic activity of TsaGH11 were investigated using beechwood xylan as the substrate. The optimum temperature for enzymatic hydrolysis of xylan by TsaGH11 was found to be 70 °C (Fig. 1A). More than 80% relative activities (RA) were observed at 40, 50, and 60 °C representing 84.1, 91.5, and 97.3% of Ras, respectively, compared to the maximum activity at 70 °C. Even at lower temperatures, such as 20 and 30 °C, TsaGH11 exhibited high activity (RA > 40%). In addition, the enzyme was active at high temperatures of 80 and 90 °C, with RAs of 52.8 and 30.2%, respectively. When the effect of pH on the enzymatic activity of TsaGH11 was investigated, the optimum pH was observed to be slightly acidic, ranging from pH 4.0 to 6.0, with no significant difference (*p* > 0.05) (Fig. 1B). At pH 7.0, enzyme activity rapidly decreased to less than 60% RA, which was further reduced to 16.3% at pH 9.0. At pH 10.0 and 11.0, the enzymatic activity of TsaGH11 was almost zero.

*T. saccharolyticum* is capable of growing under saline conditions, being classified as a halophile^19^. Accordingly, the effect of salt on the enzymatic activity of TsaGH11 was examined using NaCl concentrations of 0–2.5 M. The enzyme showed little difference in its xylan hydrolyzing efficacy when incubated under the tested saline conditions relative to that when incubated without NaCl addition (Fig. 1C). Enzyme kinetics were investigated using beechwood xylan as substrate, and the results revealed that the *K*_*m*_, *k*_*cat*_, and *k*_*cat*_/*K*_*m*_ values of 12.9 ± 1.9 mg mL^−1^, 34,015.3 ± 3714.5 s^−1^, and 2658.7 ± 195.2 mL mg^−1^ s^−1^, respectively (Fig. 1D and Table 1).

**Table 1:** Comparison of kinetic parameters of the GH family 11 xylanases towards beechwood xylan.

Enzyme	Source	*K*_*m*_ (mg ml^−1^)	*k*_*cat*_ (s^−1^)	*k*_*cat*_/*K*_*m*_ (ml mg^−1^ s^−1^)	Optimal condition	Reference
TsaGH11	*Thermoanaerobacterium saccharolyticum*	12.9	34,015.3	2658.7	pH 5.0, 70 °C	This study
NhGH11	*Nectria haematococca*	8.1	28.8	3.6	pH 6.0, 45 °C	^20^
CbGH11	*Clostridium beijerinckii*	19.1	1041.9	54.5	pH 5.0, 50 °C	^21^
rGH11XynB	*Paenibacillus* sp.	6.3^a^	64.0	10.2	pH 9.0, 45 °C	^22^
BpGH11	*Bacillus pumilus*	20	343.6	17.2	pH 6.5, 50 °C	^23^
Xyn2	*Trichoderma reesei*	4.5	89.4	19.9	pH 6.0, 50 °C	^24^

^a^The value indicates the* K*_*half*_ value of rGH11XynB obtained from the atypical sigmoidal kinetic profile.

### Cleavage pattern

**Figure 2:**
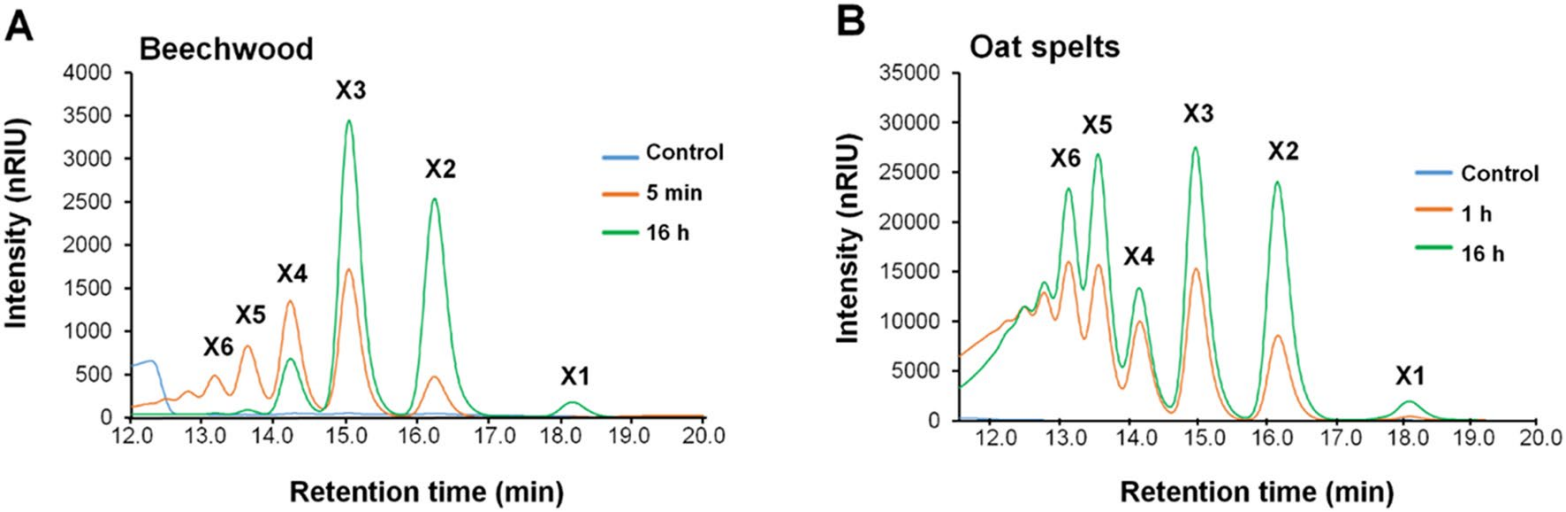
Profile of xylan hydrolysis products. Enzymatic hydrolysis was conducted using (**A**) beechwood (0.5%, *w/v*) or (**B**) oat spelts xylan (5%, *w/v*) as substrates at 70 °C and pH 5.0 (50 mM sodium acetate). The reaction products were analyzed by HPLC as described in Materials and Methods. X1–X6 represents the soluble xylose products with different DPs ranging from xylose to xylohexaose.

To better understand the xylan processing by TsaGH11, the products obtained from hydrolysis of beechwood and oat spelts xylans were examined using high-performance liquid chromatography (HPLC) analysis (Fig. 2). Based on the HPLC analysis conducted here, the product information on the presence/type/degree of substitutions is limited. Only the information on the degree of polymerization (DP) of products is known. Thus, for simplicity, the reaction products obtained from HPLC were annotated as xylose, xylobiose (X2), xylotriose (X3), xylotetraose (X4), xylopentaose (X5), and xylohexaose (X6). In the control reaction without TsaGH11, there was no detectable soluble sugar product using both substrates (data not shown). In contrast, soluble xylooligosaccharides were generated from both substrates by treatment with TsaGH11, proving its hydrolyzing activity on xylans. In the case of beechwood xylans, diverse xylooligosaccharide products, X2, X3, X4, X5, and X6, were observed after 5 min of enzymatic incubation (Fig. 2A). With further incubation (16 h), the high degree of polymerization (DP) peaks (X4–X6) decreased or disappeared, whereas increases in the peak intensity of lower DP products were observed, X2 and X3 being the major products with trace amounts of xylose (X1) (Fig. 2A). These results show the endo-type cleavage pattern of TsaGH11 towards polymeric beechwood xylan, further indicating the capability of the enzyme to hydrolyze high-DP oligosaccharides into low-DP oligosaccharides. Hydrolysis of oat spelt xylan also generated a wide range of xylooligosaccharides, but in contrast to beechwood xylan, high-DP products were not hydrolyzed into low- DP products even after longer incubation (Fig. 2B). Overall, TsaGH11 acted on the polymeric structure of both substrates to produce soluble oligosaccharides with various DPs; however, only oligosaccharides from beechwood xylan were further hydrolyzed into smaller products.

### Overall structure of TsaGH11

To better understand the molecular function of TsaGH11, a crystallography study was performed and this initially determined the 1.4 Å crystal structure of TsaGH11 at cryogenic temperatures (Table 2). The TsaGH11 crystals belong to the tetragonal space group P4_3_2_1_2 and contain two molecules in an asymmetric unit. The R_work_ and R_free_ of the final models of TsaGH11 were 16.55 and 18.97, respectively. The electron density map of the crystal structure of TsaGH11 was clearly observed for all amino acids.

**Table 2:** Data collection and refinement statistics of TsaGH11.

Data collection	Cryo-TsaGH11	RT-TsaGH11
Temperature (K)	100	300
Exposure time (s)	1	0.1
Collected images	360	
Hit images		32,250
Indexed images	360	32,203
Diffraction pattern	360	65,986
Space group	P4_3_2_1_2	P4_3_2_1_2
Cell dimensions (Å) a, b, c	73.11, 73.11, 165.42	74.47, 74.47, 167.74
Resolution (Å)	50.0–1.50 (1.53–1.50)	172.4–1.75 (1.78–1.75)
No. of reflections	72,944 (3573)	49,353 (4815)
Completeness	99.9 (99.5)	100.0 (100.0)
Redundancy	19.1 (7.9)	775.8 (415.3)
I/σ(I)	37.22 (2.00)	12.73 (2.61)
CC1/2	0.999 (0.624)	0.9921 (0.857)
CC*	1.000 (0.877)	0.9965 (0.967)
*R*_*split*_^a^		7.16 (37.46)
Refinement		
Resolution (Å)	49.39–1.50	68.06–1.75
*R*_*work*_ (%)^b^	16.55	23.31
*R*_*free*_ (%)^c^	18.97	29.29
B-factor (Averaged)		
Protein (chain A/B)	18.14/21.80	23.06/36.42
Waters	37.14	49.55
R.m.s deviations		
Bond lengths (Å)	0.014	0.012
Bond angles (°)	1.811	1.628
Ramachandran plot (%)		
Favored	98.1	96.0
Allowed	1.9	3.7
Disallowed		0.3

The highest resolution shell is shown in parentheses.

^a^*R*_*split*_ = $$\left(1/\sqrt{2}\right)\bullet \frac{\sum_{hkl}\left|{I}_{hkl}^{even}-{I}_{hkl}^{odd}\right|}{\frac{1}{2}\left|{I}_{hkl}^{even}-{I}_{hkl}^{odd}\right|}$$.

^b^*R*_work_ = Σ||*F*_obs_| −|*F*_calc_||/Σ|*F*_obs_|, where *F*_obs_ and *F*_calc_ are the observed and calculated structure factor amplitudes, respectively.

^c^*R*_free_ was calculated as R_work_ using a randomly selected subset (9.47%) of unique reflections not used for structure refinement.

**Figure 3:**
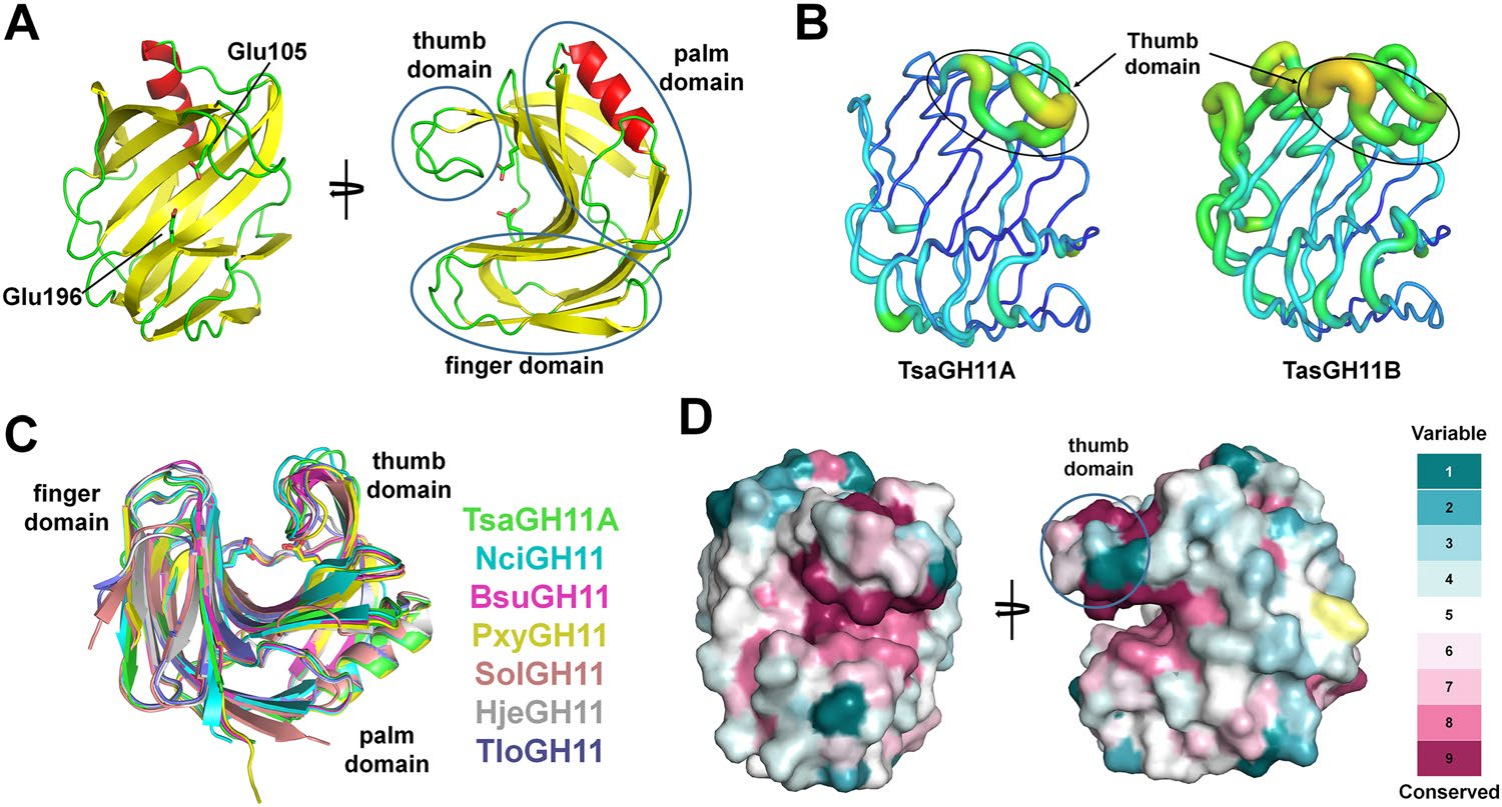
Overall structure of TsaGH11. (**A**) Crystal structure of TsaGH11 forming the β-jelly roll fold consisting of thumb-, palm- and finger-domains. (**B**) B-factor putty representation of molecules A and B of TsaGH11 at cryogenic temperature. (**C**) Superimposition and TsaGH11 with GH11 from *Niallia circulans* (NciGH11, PDB code: 1XNB), *Bacillus subtilis* (BsuGH11, 2B45), *Paenibacillus xylanivorans* (PxyGH11, 7KV0), *Streptomyces olivaceoviridis* (SolGH11, 7DFN), *Hypocrea jecorina* (HjeGH11, 4HK8) and *Trichoderma longibrachiatum* (TloGH11, 3AKR). (**D**) Conservation surface of TsaGH11 among the GH11 family.

TsaGH11 consists of 12 β-strands and one α-helix and exhibits a canonical β-jelly roll fold (Fig. 3A), which is typical of the GH11 family. The structure of TsaGH11 resembles that of the right hand, where the β-strand forms the finger and palm domains constituting the active site cleft (Fig. 3A). Accordingly, access to the active site cleft is regulated by the movement of flexible β-turn structures termed the “thumb” region^25^. In the asymmetric unit, two TsaGH11 molecules (named TsaGH11A and TsaGH11B) form a twofold symmetric antiparallel β-sheet by the main chain interaction of β1-strands (Supplementary Fig. S1), which is a crystallographic artifact because the TsaGH11 was eluted as a monomer in solution. The superimposition of two TsaGH11 molecules has root-mean-square (r. m. s.) deviations of 0.231 Å. The overall B-factor values of TsaGH11A and TsaGH11B were 18.14 and 21.80 Å^2^, respectively. B-factor putty representation showed that the two TsaGH11 molecules in the asymmetric unit showed distinct molecular flexibility (Fig. 3B). In crystal packing, the finger and palm domains of TsaGH11A interact with neighboring molecules involved in protein packing in the crystal lattice (Supplementary Fig. S2). Meanwhile, in TsaGH11B, the finger domain interacts with neighboring molecules in the crystal lattice, whereas most of the thumb and palm domains are exposed to solvent channels in the crystal lattice (Supplementary Fig. S2). Accordingly, the finger and palm domains of TsaGH11B can be more flexible than the TsaGH11A molecule due to the crystal packing effect. However, the average B factor value of the finger domains of TsaGH11A and TsaGH11B were 26.07 and 29.72 Å^2^, respectively, indicating no significant flexibility of the finger domain of TsaGH11 at cryogenic temperatures.

A structural homology search showed that TsaGH11 has structural similarity to *endo*-1,4-*β*-xylanases from *Niallia circulans* (PDB code, 1XNB; Z-score, 35.9; r.m.s. deviation, 0.6 Å; sequence identity, 78%), *Bacillus subtilis* (2B45, 35.8, 0.6 Å, 78%), *Paenibacillus xylanivorans* (7KV0, 35.6, 0.6 Å, 76%), *Streptomyces olivaceoviridis* (7DFN, 30.6, 0.9 Å, 65%), *Hypocrea jecorina* (4HK8, 30.5, 1.0 Å, 53%), and *Trichoderma longibrachiatum* (3AKR, 30.4, 1.1 Å, 54%) (Fig. 3C). Sequence alignment and surface conservation showed that catalytic residues (numbered according to TsaGH11: Glu105 and Glu198) and substrate-binding residues (numbered according to TsaGH11: Trp36, Asn62, Asn90, Tyr92, Tyr96, Trp98, Tyr115, Pro117, Arg139, Gln153, Trp155, Tyr192, and Try200) in the substrate-binding cleft were highly conserved (Fig. 3D and Supplementary Fig. S3). This result indicates that the substrate recognition and catalytic mechanisms of TsaGH11 are similar to those of other GH11 homologs. Superimposition of the structures of TsaGH11 and GH11 homologs indicated that the thumb domains of GH11 show an intrinsic conformation, whereas the finger and palm domains show high structural similarity.

### Substrate binding cleft and active site of TsaGH11

**Figure 4:**
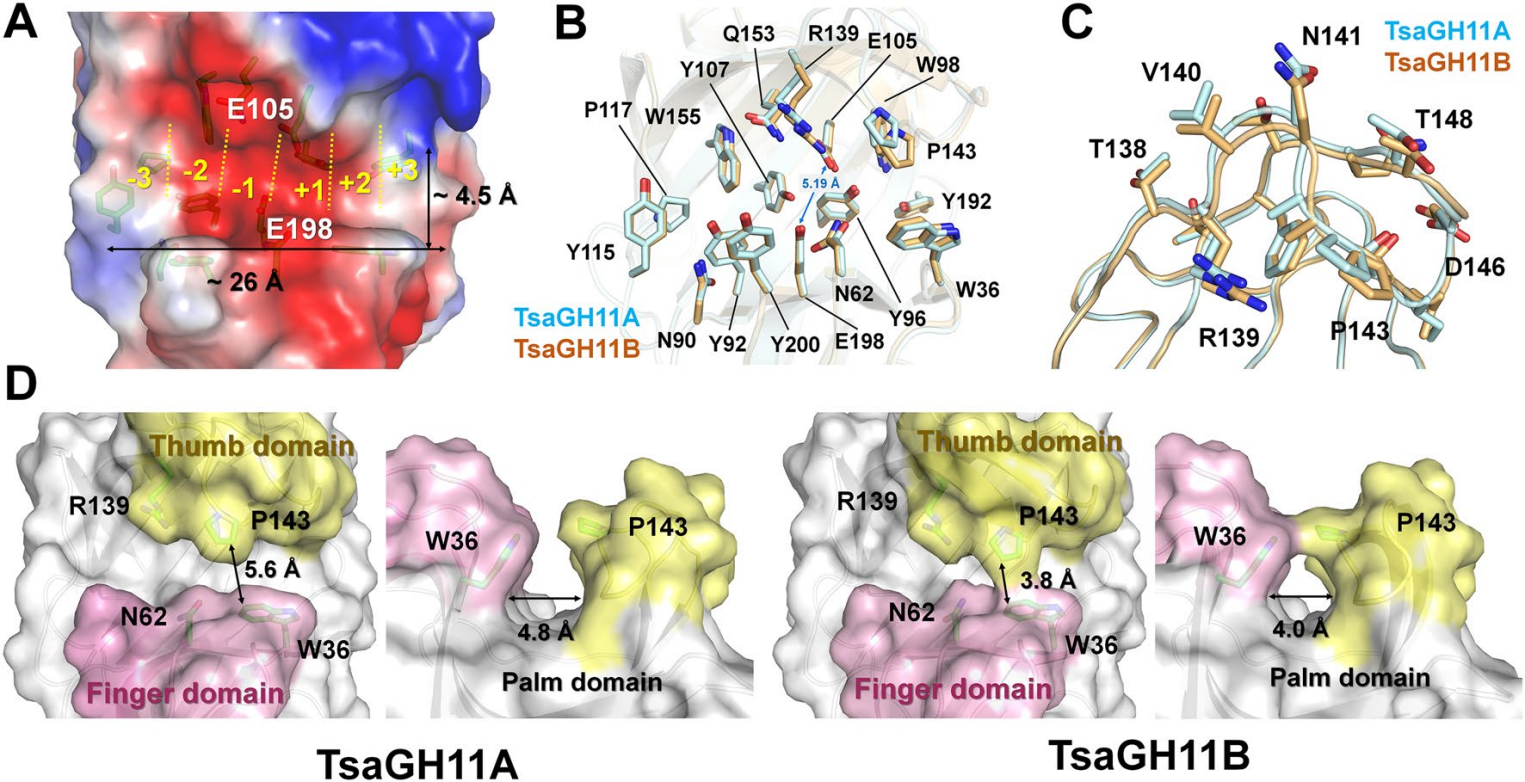
Substrate binding cleft of TsaGH11 at cryogenic temperatures. (**A**) Electrostatic surface representation of the substrate binding cleft of TsaGH11. Superimposition of (**B**) catalytic and substrate binding residues, and (**C**) thumb domains of TsaGH11A (cyan) and TsaGH11B (orange). (**D**) TsaGH11A and TsaGH11B surface structures exhibited open and close conformations of the thumb domain, respectively.

The negatively charged surface of the substrate-binding cleft of TsaGH11 is located between the palm and finger domains with dimensions of 14 Å (depth) × 4.5 Å (width) × 26 Å (length) Å (Fig. 4A) and contains six subsites. As TsaGH11 has a cylindrical-shaped substrate-binding cleft, it can accommodate a linear xylan backbone (Fig. 4A). The catalytic residues Glu105 (nucleophile) and Glu198 (acid–base) are located in the middle of the cleft and are positioned between the + 1 and − 1 subsites. The distance between the side chains of the two catalytic glutamate residues was 5.19 Å (Fig. 4B). These two catalytic glutamate residues lead to the cleavage of xylosidic linkages, with net retention of the anomeric configuration^26^. The substrate-binding cleft is surrounded by aromatic amino acids Trp36, Tyr92, Trp98, Tyr115, Trp155, and Tyr200 (Fig. 4B), the overall features of which are similar to other GH11s^20^. π…π and C–H…π interactions were observed between the aromatic side chains (Trp98-Phe151 and Tyr107-Trp155) in the substrate binding cleft of TsaGH11 (Fig. 4B), which was consistent with observations of other GH11s, suggesting that it contributes to thermostability^20^. The thumb-forming Asn141, Gly147, and Thr150 residues are conserved as with other GH11s (Supplementary Fig. S3), partially covering the active site cleft of TsaGH11. Interestingly, the superimposition of TsaGH11A and TsaGH11B showed a large difference in conformation of the thumb domains, from Ala137 to Phe150 (Fig. 4C and Supplementary Fig. S4). The thumb domain of TsaGH11B was shifted toward the active site direction when compared with the position of the thumb domain of TsaGH11A (Fig. 4C). The largest positional change was in the Asn141 residue, with a distance of 1.8 Å between the Cα atoms of two TsaGH11 molecules. Meanwhile, other residues involved in substrate binding or catalytic activity showed almost similar conformations, except for the slightly different conformations of the side chains of Trp36 (distance: 0.49 Å), Asp38 (0.44 Å), and Trp98 (0.69 Å). This result indicates that the conformational change in the thumb domain of TsaGH11 may have little effect on the conformation changes in the neighbor residues around the active and substrate-binding sites. The difference in conformation of the thumb domain could affect the substrate binding cleft of TsaGH11. In the surface structure of TsaGH11, TsaGH11A exhibited an open conformation between the finger and thumb domains, whereas TsaGH11B showed a closed conformation between the thumb and finger domains (Fig. 4D). A previous study proposed that partial closure of the cleft by the movement of the thumb domain occurred via ligand binding^27^. In contrast, our experimental results showed that the thumb domain of TsaGH11 could represent both open and partially closed substrate-binding clefts in the absence of substrate binding. The closest distances between Pro440 in the thumb domain and Phe225 residues in the finger domains of TsaGH11A and TsaGH11B were 5.6 and 3.8 Å, respectively (Fig. 4D).

According to the different conformations of the thumb region of TsaGH11s, the width of the catalytic cleft of TsaGH11A and TsaGH11B differed by approximately 4.8 and 4.0 Å, respectively (Fig. 4D), consistent with a previous suggestion that the precise position of the thumb domain of GH11 determines the width of the catalytic cleft^25^.

### Room-temperature structure of TsaGH11

The molecular flexibility of room-temperature structures are indicative of the relatively reliable functional structures of macromolecules compared with that at cryogenic temperatures^28,29^. To better understand the molecular flexibility of TsaGH11, we determined its room-temperature structure (RT-TsaGH11) using serial synchrotron crystallography. TsaGH11 crystals grown at pH 4.5 were harvested and delivered to the X-ray interaction point using a nylon-mesh-based sample holder (Supplementary Fig. S5). A total of 32,250 images were collected, and 65,986 indexed patterns were obtained from 32,203 hit images containing Bragg peaks (Supplementary Fig. S6). The indexing and multi-crystal hit rates were approximately 99.85% and 104.89%, respectively. The space group and unit cell parameters of RT-TsaGH11 were similar to those of the cryogenic structure of TsaGH11 (Table 2). The indexed images were processed up to 1.75 Å and showed completeness, redundancy, signal-to-noise ratio (SNR), CC*, and R_split_ values of 100, 775.8, 0.9965, 12.73, and 7.16, respectively (Table 2). The diffraction data of RT-TsaGH11 was validated by Xtriage, and no issues were found in the quality of the processed data. The phase was successfully solved by molecular replacement using the cryogenic structure of TsaGH11. The R_work_ and R_free_ of the final model structure of RT-TsaGH11 were 23.31 and 29.29, respectively. These R-values are reliable but are much higher than those of cryo-TsaGH11 owing to the relative flexibility of the TsaGH11B molecule at room temperature.

**Figure 5:**
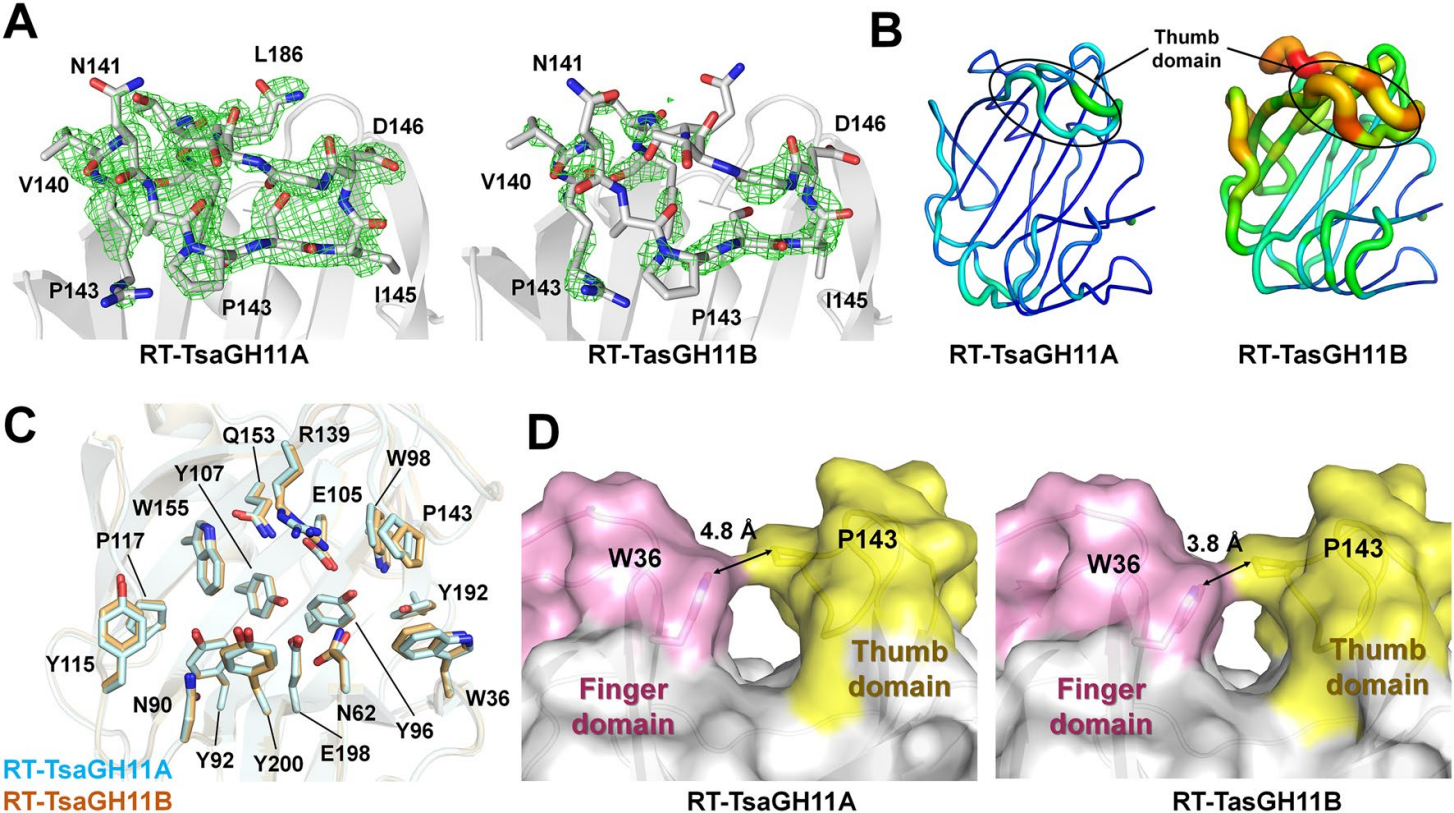
Room-temperature structure of TsaGH11 obtained by serial synchrotron crystallography. (**A**) F_o_–F_c_ omit electron density map (green mesh, 3σ) of the finger domains of RT-TsaGH11. (**B**) B-factor putty representation of RT-TsaGH11. (**C**) Superimposition of the substrate binding cleft of RT-TsaGH11 molecules. (**D**) Surface structure of RT-TsaGH11.

In RT-TsaGH11, the electron density map corresponding to the TsaGH11A molecule was clear for all the amino acids (Fig. 5A). In RT-TsaGH11B, the electron density map corresponding to the finger domain was clearly ordered; however, the thumb domain of RT-TsaGH11B was largely disordered (Fig. 5A). The main chains of the thumb and palm domains of RT-TsaGH11B could be traced using the cryogenic structure of TsaGH11 at a low contour level of the electron density map, but the accuracy of the position of the side chains of the thumb domain of RT-TsaGH11B was not high, indicating increase in flexibility of TsaGH11B at room temperature (refer to Discussion). The difference in flexibility between these two RT-TsaGH11s affects the packing of crystals as well as the room temperature. Since the thumb domain of TsaGH11A is involved in crystal packing, this domain has a relatively rigid conformation, whereas the thumb domain of RT-TsaGH11B exposed to the solvent can dynamically become disordered at room temperature. The B-factor values of molecules A and B of the final model structure of RT-TsaGH11 were 23.06 and 36.42 Å^2^, respectively (Fig. 5B), indicating that the B-factor of RT-TsaGH11B is approximately 1.67-fold higher than that of RT-TsaGH11A. Superimposition of RT-TsaGH11 molecules showed a slightly different conformation of the side chain of substrate binding residues Tyr115, Pro143, Tyr192, and Tyr200 as well as catalytic residue Glu198 (Fig. 5C). The closest distances between Pro143 of the thumb domain and W36 of the finger domain, from RT-TsaGH11A and RT-TsaGH11B, were 4.84 and 3.86 Å, respectively (Fig. 5D). The surface structure of both RT-TsaGH11A and RT-TsaGH11B showed a closed conformation.

### Comparison of RT-TsaGH11 and Cryo-TsaGH11

**Figure 6:**
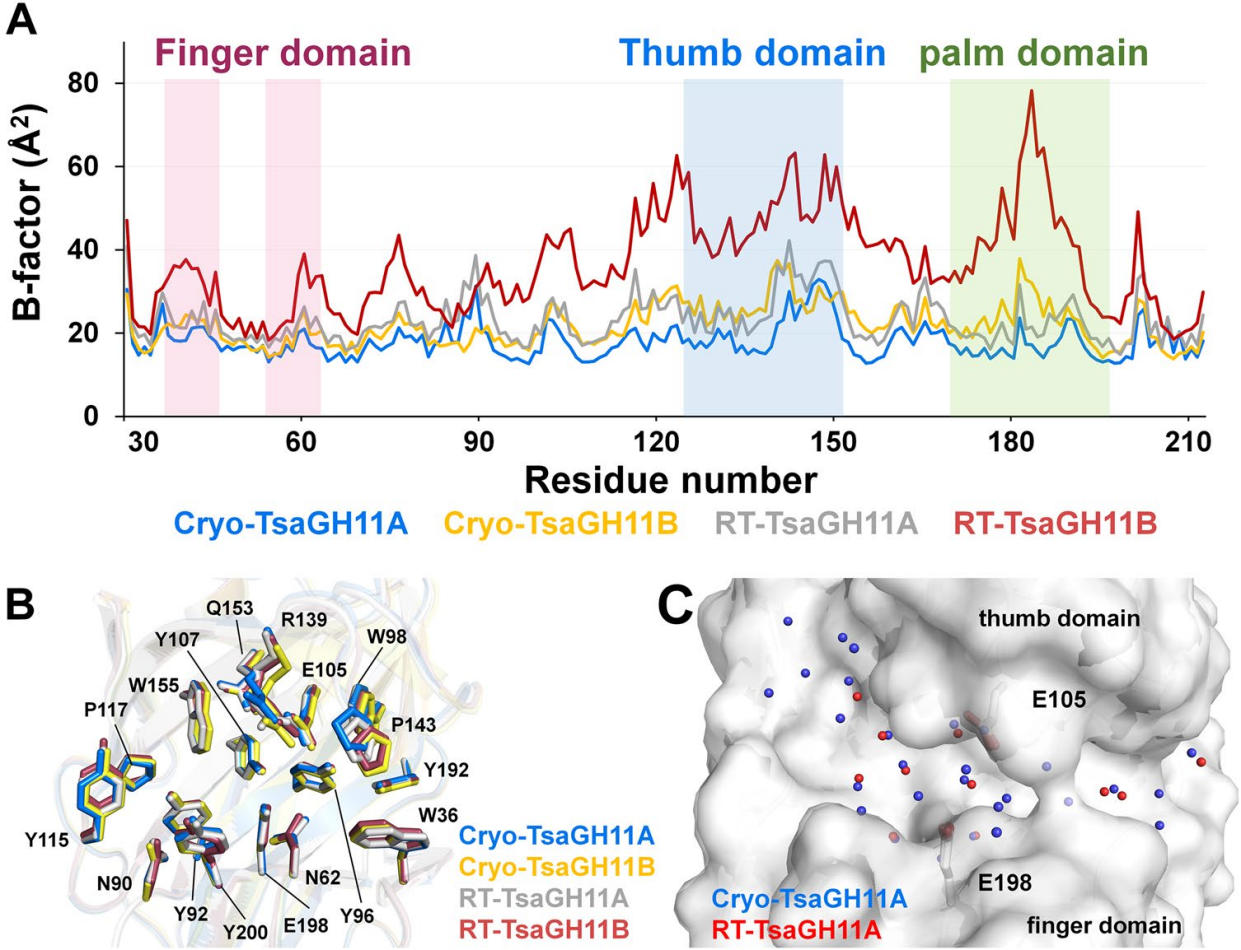
Comparison of the crystal structures of RT- and Cryo-TsaGH1. (**A**) Profile of B-factor values of room-temperature and cryogenic structures of TsaGH11. (**B**) Superimposition of room-temperature and cryogenic structures of TsaGH11. (**C**) Superimposition of water molecules on substrate binding cleft of RT-and Cryo-TsaGH11A.

To understand the relationship between structure and temperature, crystal structures of TsaGH11 at room and cryogenic temperatures (named Cryo-TsaGH11) were compared. The profile of temperature factor TsaGH11 showed that the overall B-factor of RT-TsaGH11A, Cryo-TsaGH11A, and Cryo-TsaGH11B are similar, in the range of 18.14–23.06 Å^2^, whereas the B-factor value of RT-TsaGH11B is approximately 1.5-fold larger than other TsaGH11 molecules (Fig. 6A). In particular, the average B-factors of the thumb domain (R139-F151) of RT-TsaGH11A, RT-TsaGH11B, Cryo-TsaGH11A, and Cryo-TsaGH11B were 32.24, 57.18, 24.87, and 28.17 Å^2^, respectively, indicating that the largely disordered thumb domain of TsaGH11B was significantly flexible than in other molecules. In addition, an α-helix region (Lys180-Asn185) on the palm domain of TsaGH11B also showed higher B-factor values than the other molecule. Superimposition of the RT- and Cryo-TsaGH11A showed that the positions of the highly conserved Tyr96, Trp98, Glu105, and Tyr192 residues in the palm domain were almost identical, whereas the position of the side chain of Tyr115, Tyr200 and Gln201 in the thumb domain were relatively different (Fig. 6B and Supplementary Fig. S7). All TsaGH11 molecules showed a positional difference of the thumb domain (Fig. 6B). The thumb domain of RT-TsaGH11A moved by 0.8 Å toward the finger domain compared with TsaGH11A at cryogenic temperature. Accordingly, the interspace between the finger and thumb domains of TsaGH11 at room temperature was slightly narrower than that of cryogenic TsaGH11. Water molecules are important in enzyme reactions and stabilize the charged residues in macromolecules^30^. The number of built water molecules of RT- and Cryo-TsaGH11 were 161 and 472, respectively (Supplementary Fig. S8), indicating that the water molecules have more mobility at room temperature. In the active site cleft, few water molecules were observed in the substrate-binding cleft of RT-TsaGH11, whereas a water-mediated hydrogen bond network was observed in the substrate-binding cleft of Cryo-TsaGH11A (Fig. 6C). This result indicates that the water molecules in the substrate-binding site of TsaGH11 are relatively dynamic at room temperature. The superimposition of the TsaGH11A molecule and the position of water molecules are slightly different in the substrate binding cleft.

## Discussion

Xylanases are essential and desirable targets for lignocellulose-based biotechnological applications. Here, we report the molecular properties and crystal structure of the TsaGH11 enzyme from the hemicellulose-degrading thermophilic anaerobe *T. saccharolyticum*, a widely applied strain for industrial biomass degradation^31^. The predicted molecular weight of TsaGH11 was approximately 23 kDa. The optimal temperature and pH for the enzyme activity of TsaGH11 were 70 °C and pH 4.0–5.0, respectively, which is consistent with the growth conditions (30–66 °C and pH 3.85–6.35) of *T. saccharolyticum*^18^, which is capable of surviving at elevated temperatures (up to 70 °C) and low pH. As the sequence of native TsaGH11 contains a signal peptide, we consider that mature TsaGH11 secreted out of the cell can have stable activity at high temperatures and acidic pH, similar to the *T. saccharolyticum*’s natural growth conditions. The thermophilic property of TsaGH11 is advantageous over its common mesophilic counterparts when applied in bioprocesses that frequently involve harsh conditions. Normally, enzymes originating from thermophilic organisms tend to be heat-resistant. Indeed, other lignocellulolytic enzymes from *T. saccharolyticum* also showed thermophilicity, although their optimal temperatures differ. For instance, the endo-xylanases XynA^32^ and XynFCB^33^ are maximally active at 70–73 °C and 63 °C, respectively, while the β-glucosidase TsaBgl^34^ exhibits optimal activity at 55 °C.

Xylanases from bacterial sources generally have maximum activity at neutral or alkaline pH, whereas their fungal counterparts usually require acidic conditions^35^. Interestingly, TsaGH11, which is of bacterial origin, prefers a slightly acidic pH similar to another bacterial xylanase from *Paenibacillus macerans* IIPSP3, which showed optimal activity at pH 4.5^36^. This property can benefit various industrial applications requiring acidic conditions, such as fruit juice clarification and animal feed formulation. As a halophile, *T. saccharolyticum* can be a source of halophilic enzymes whose activity is often stimulated under high salt concentrations. For example, the activity of a GH10 xylanase from *T. saccharolyticum* (XynA) is enhanced in the presence of 0–0.5 M NaCl^32^. However, TsaGH11’s activity did not increase under saline conditions but was maintained constantly at 0–2.5 M NaCl concentrations. This result indicates that TsaGH11 is not a strict halophilic enzyme, differing from the results reported for XynA from the same organism. Nevertheless, TsaGH11 did not lose its function even at high concentrations of NaCl (2.5 M). This property is particularly advantageous in food bioprocesses that often involve hyper-saline conditions.

TsaGH11 enabled the hydrolysis of both beechwood and oat spelt xylans but preferred beechwood xylan. Hardwood-derived xylans, including beechwood, termed glucuronoxylan, are made up of a linear xylose backbone with a β-1,4-glycosidic linkage substituted by 4-*O*-methyl-α-glucuronic acid with α-1,2-linkage^37^. In the case of xylan from cereals, including oat spelt, termed arabinoxylan, the xylan backbone is substituted with arabinose as the side chain. The degrees of substitution of beechwood and oat spelts xylan used in this study were estimated to be approximately 10% glucuronic acid and 17.5% arabinose, respectively. The higher specific activity of TsaGH11 towards beechwood xylan may be explained by its less complex structure relative to that of oat spelt xylan. In addition, TsaGH11 showed a different product profile towards the two substrates, in which xylooligosaccharides generated from beechwood were hydrolyzed whereas the same was not true with those generated from oat spelts. Such a phenomenon could be associated with differences in the structures of the two substrates (type and degree of substitution). For example, enzyme access might be more restricted by the branched oligosaccharides of oat spelt xylan, which could be generated with a higher frequency due to its higher degree of substitution.

Compared with other GH11 xylanases from *Nectria haematococca*^20^, *Clostridium beijerinckii*^38^, *Paenibacillus* sp.^39^, *Bacillus pumilus*^40^, and *Trichoderma reesei*^41^*,* using beechwood xylan as a substrate, the *K*_*m*_ value of TsaGH11 was higher, indicating its low affinity for the substrate (Table 1). However, the *k*_*cat*_ value, which represents the turnover number of an enzyme, was much higher, and in turn, the catalytic efficiency (*k*_*cat*_/*K*_*m*_*)* was also higher by 10^2^–10^3^ orders of magnitude. Overall, these results demonstrate the excellent catalytic performance of TsaGH11.

TsaGH11 exhibits a typical β-jelly roll fold and contains highly conserved catalytic and substrate-binding residues similar to other GH11 family members. Since the xylanase activity of TsaGH11 at pH 4.5 is at a maximum (Fig. 1), the crystal structures of TsaGH11 are associated with relatively high activity. The crystal structures determined in this study exhibited two distinct conformations of the thumb domains of TsaGH11 due to the crystal packing effect. These results indicate that the flexibility of the thumb domain of TsaGH11 is affected by interactions with neighboring molecules. As the TsaGH11 molecule does not interact with other TsaGH11 molecules in solution or in vivo, the flexible conformation of the thumb domain of TsaGH11B exposed to the solvent area in the crystal lattice is closer to the native conformation of TsaGH11 than the rigid conformation of TsaGH11A.

Because the conformation of the finger and thumb domains of GH11 is correlated with the molecular mechanism of xylanase GH11 enzymatic activity, many structural and computational studies have analyzed the dynamics of these domains^42^. However, the previously determined crystal structures of GH11 were determined by conventional cryo-crystallography, which has experimental limitations in terms of cryogenic temperatures and radiation damage. In particular, cryogenic structures have less structural flexibility than room-temperature structures^28,29^. To better understand the molecular flexibility of TsaGH11, we determined the crystal structure of TsaGH11 using serial crystallography, which resembles the room-temperature structure while minimizing radiation damage. The overall temperature factor value of the room-temperature structure of TsaGH11 is relatively higher than that of the cryogenic structure of TsaGH11, indicating that temperature is a factor for the molecular fluctuation of TsaGH11. In particular, the electron density map corresponding to the thumb domains of RT-TsaGH11B exposed to the solvent channel in the crystal lattice was disordered. This result clearly indicates that the thumb domain of TsaGH11 is highly dynamic rather than favoring a specific fixed conformation at room temperature. Various computational analyses have attempted to understand the flexibility of the finger and thumb domains of GH11^42,43^. Our experimental evidence of the disordered thumb domain in RT-TsaGH11B will provide insights into understanding the molecular flexibility and function at the active site of GH11. Meanwhile, our biochemical results showed that the xylanase activity of TsaGH11 at room temperature was approximately 70% of the relative activity compared with that at its optimal temperature (70 °C). Accordingly, we expected that the flexible domain of TsaGH11 might be more dynamic at optimal temperature. On the other hand, RT-TsaGH11A showed a slightly higher B-factor compared with that of Cryo-TsaGH11A but still had a rigid conformation, which was likely due to the interaction with surrounding molecules via crystal packing. This indicates that, even at room temperature, the flexibility of a protein can be limited by the packing effect of the protein in the crystal lattice. These crystallographic results can serve as a good example of the effect of crystal packing on the structural flexibility of proteins. The disordered electron density map of the thumb domain of RT-TsaGH11B can provide insights into the structural dynamics of TsaGH11 as well as the GH11 family and enzyme engineering.

The high *k*_*cat*_* value* of TsaGH11 implies that the enzyme is highly efficient in catalyzing substrate conversion when saturated with substrate. TsaGH11 may have unique structural features that facilitate efficient catalysis and product release, allowing it to process substrates at a higher rate (that is, high *k*_*cat*_) than other GH11s. This could be related to the geometry of active site and binding cleft, substrate binding subsites, and loop regions, which likely results in specific catalytic mechanism and enzyme–substrate interaction. However, it is difficult to discover the exact structure–function relationship in this study since the structure of TsaGH11 was determined at RT. It would be interesting if structural features contributing to the superior *k*_*cat*_ value of TsaGH11 are elucidated through structure determination at its optimal temperature (70 °C).

To conclude, TsaGH11's superior catalytic efficiency with high industrial value can be useful for various applications. Furthermore, our structural study will contribute to broadening our knowledge of the structural flexibility of the thumb domain of the GH11 family.

## Materials and methods

### Expression and purification of TsaGH11

The signal peptide of TsaGH11 was analyzed using SignalP-5.0^44^. For expression in *Escherichia coli*, a codon-optimized TsaGH11 (UniProt: I3VTR8) gene, excluding the signal peptide, was synthesized with the addition of an N-terminal hexahistidine for affinity purification. The synthesized gene was cloned into a pBT7 vector (Bioneer, Daejeon, Korea) and transformed into *E. coli* BL21 (DE3) strain. Cells were grown at 37 °C in LB medium with 50 mg mL^−1^ ampicillin until the OD_600nm_ reached 0.4–0.8. Protein expression was induced using 0.5 mM isopropyl-D-1-thiogalactopyranoside, and the cells were incubated further at 18 °C for 18 h. Thereafter, the cells were harvested by centrifugation at 4500 × *g* for 20 min. The cell pellet was resuspended in a lysis buffer containing 50 mM Tris–HCl, pH 8.0, 200 mM NaCl, and 20 mM imidazole. Next, the cells were disrupted by sonication on ice, and the cell debris was removed by centrifugation at 18,894 × *g* for 30 min. The supernatant was then loaded onto an affinity column containing the Ni–NTA resin (Qiagen, Valencia, CA, USA). The resin was washed with lysis buffer, and the protein was eluted with a buffer containing 50 mM Tris–HCl, pH 8.0, 200 mM NaCl and 300 mM imidazole. The N-terminal hexahistidine-tag of TsaGH11 was cleaved by addition of thrombin (Sigma-Aldrich, St. Louis, MO, USA) at 25 °C for > 18 h. Subsequently, the protein was concentrated using a Centricon filter (Merck Millipore, Burlington, MA, USA; cut-off: 30 kDa) and loaded onto a Sephacryl S-100 size exclusion chromatography column (GE Healthcare, Chicago, IL, USA) equilibrated with 10 mM Tris–HCl, pH 8.0 buffer containing 200 mM NaCl. The fractions from the main elution peak measured by UV absorption at 280 nm during size exclusion chromatography were used for the enzyme assay. Other protein fractions were collected and concentrated to 20 mg mL^−1^ for crystallization. Protein purity was assessed using 15% SDS-PAGE, and protein concentration was determined using the Bradford assay.

### Enzyme assay of TsaGH11

Unless otherwise stated, the hydrolytic activity of TsaGH11 was investigated by incubating the purified enzyme (1.75 nM) with 0.5% (*w/v*) xylan substrate from beechwood (Sigma-Aldrich) at 70 °C and pH 5.0 (50 mM sodium acetate buffer) for 1 h in a total volume of 100 µL. To investigate the effect of temperature on the activity of TsaGH11, the enzyme assay was conducted at different temperatures ranging from 4 to 90 °C in 50 mM sodium acetate buffer, pH 5.0. To investigate the effect of pH on the TsaGH11 activity, the enzyme assay was conducted at different pH values, ranging from 4.0 to 11.0 at 70 °C. The buffers used were 50 mM sodium acetate (pH 4.0–5.0), 50 mM sodium phosphate (pH 6.0–8.0), 50 mM Tris–HCl (pH 8.0–9.0), and 50 mM glycine–NaOH (pH 10.0–11.0). After taking a 40 μL aliquot, the enzyme reaction was stopped by adding 80 μL 3,5-dinitrosalicylic acid (DNS) reagent, which was then boiled at 95 °C for 5 min for color development. After centrifugation at 13,000 × *g* for 10 min at 25 °C, the supernatant (100 μL) containing released sugars was collected and spectroscopically measured at 540 nm using a Synergy microplate reader (BioTek, Winooski, VT, USA). The concentration of the sugar products was calculated from the calibration curve constructed using xylose as the standard. One unit (U) of enzyme activity was defined as the amount of TsaGH11 required to produce 1 µmol of xylose equivalent from 0.5% (*w/v*) xylan per minute under specified conditions.

### Enzyme kinetics

Kinetic parameters (*K*_m_ and *k*_cat_) were determined by conducting enzyme assays with 1.75 nM of TsaGH11 with various concentrations (0–40 mg mL^−1^) of xylan from beechwood under optimal conditions (pH 5.0, 70 °C) for 10 min. Data fitting was performed using the Michaelis–Menten equation in SigmaPlot 12.3 software (Systat Software, Erkrath, Germany).

### Product analysis

The sugar products were obtained from enzymatic hydrolysis of the xylan substrates from beechwood (0.5%, *w/v*) or oat spelts (5%, *w/v*) under optimal conditions (pH 5.0, 70 °C) for the indicated incubation times. After centrifugation at 13,000 × *g* for 10 min, only supernatants containing soluble sugar products were analyzed by HPLC (Agilent 1100; Agilent Technologies, Waldbronn, Germany) as previously described^45^. Briefly, to separate and detect the reaction products, a Shodex KS-802 column (Showa Denko, Tokyo, Japan) set at 80 °C and a refractive index detector (Agilent Technologies) were used, respectively. Distilled water was applied as the mobile phase in the column at a flow rate of 0.5 mL min^−1^. Standard solutions (1 mM) of xylooligosaccharides (X1–X6) (Megazyme, Bray, Wicklow, Ireland) were analyzed together with the reaction samples for product identification.

### Protein crystallization

The crystallization conditions of TsaGH11 (~ 20 mg mL^−1^) were screened using the sitting drop vapor diffusion method at 22 °C using commercially available crystallization kits (Crystal Screen Kit, Index, and PEG/Ions [Hampton Research, Aliso Viejo, CA, USA]). The protein solution (0.5 μL) was mixed with crystallization solutions (0.5 μL) and equilibrated with a reservoir solution (60 μL). Microcrystals were obtained with a reservoir solution containing 0.1 M sodium acetate, pH 4.6 and 4.0 M ammonium acetate. Suitable crystals for X-ray diffraction data collection were further optimized using the hanging-drop vapor diffusion method at 22 °C by scaling up of the crystallization sample volume. TsaGH11 crystals were obtained within 1 month by mixing 2 µL of the protein solution with 2 µL of the reservoir solution.

### Cryogenic X-ray diffraction data

X-ray diffraction data were collected on Beamline 11C at Pohang Light Source II (PLS-II; Pohang, Korea). The cryoprotectant solution was prepared by mixing 7.5 µL of the reservoir solution and 2.5 µL of 100% (*v/v*) ethylene glycol. The crystals were transferred into a cryoprotectant solution for 5–10 s and then mounted on a goniometer in a liquid nitrogen stream at 100 K. Diffraction images were processed using HKL2000^46^.

### Serial synchrotron crystallography

Using a pipette, TsaGH11 crystals were harvested from crystallization drops (total volume: ~ 200 μL) on a siliconized cover slide from a 24-well VDX plate (Hampton Research, Aliso Viejo, CA, USA). Next, the TsaGH11 crystals were transferred to a 1.5-mL microcentrifuge tube and incubated at room temperature for 6 h to settle the crystals to the bottom, and then the supernatant (100 μL) was discarded. A nylon mesh and enclosed film (NAM)-based sample holder was used for sample delivery^47^. Briefly, 25 μL of the crystal suspension was loaded onto the nylon mesh-based sample holder^48^, which was enclosed by a polyimide film (25 μm) to prevent dehydration of the crystal suspension. During X-ray data collection, the sample holder containing the TsaGH11 crystals was raster scanned at 50-μm intervals in the vertical and horizontal directions. Raster scanning was done at 100 ms exposure with 0.022° oscillation. Images containing the Bragg peaks were filtered using Cheetah^49^. Diffraction patterns were processed using CrystFEL^50^ with the XGANDALF^51^ indexing algorithm.

### Crystal structure determination and analysis

The phasing problem was solved by molecular replacement with MOLREP^52^. The search model was generated using Alphafold2^53^. The model of the structure was built using the COOT^54^ program. Model refinement was performed using REFMAC5^21^. The geometry of the final model structures was evaluated using MolProbity^22^. The protein structures were visualized and analyzed using PyMOL (DeLano Scientific LLC, San Carlos, CA, USA). Structure-based sequence alignments were generated using Clustal-Omega and ESPript^23^. Homologous structures were searched for using the DALI server^24^.

### Supplementary Information

Supplementary Figures.

## Data Availability

The structure factors and coordinates were deposited in the Protein Data Bank under accession codes 8IH0 (Cryo-TsaGH11, https://doi.org/10.2210/pdb8IH0/pdb) and 8IH1 (RT-TsaGH11, https://doi.org/10.2210/pdb8IH1/pdb). Diffraction images of RT-TsaGH11 have been deposited in Zenodo under the accession https://doi.org/10.5281/zenodo.7101471 and https://doi.org/10.5281/zenodo.7106300.

## References

[1] Fatma S (2018). Lignocellulosic biomass: A sustainable bioenergy source for the future. Protein Pept. Lett..

[2] Ambaye TG (2021). Emerging technologies for biofuel production: A critical review on recent progress, challenges and perspectives. J. Environ. Manag..

[3] Yadav KK (2021). Review on evaluation of renewable bioenergy potential for sustainable development: Bright future in energy practice in India. ACS Sustain. Chem. Eng..

[4] Guragain YN, Vadlani PV (2021). Renewable biomass utilization: A way forward to establish sustainable chemical and processing industries. Clean Technol..

[5] Gray KA, Zhao L, Emptage M (2006). Bioethanol. Curr. Opin. Chem. Biol..

[6] Kennes D, Abubackar HN, Diaz M, Veiga MC, Kennes C (2016). Bioethanol production from biomass: Carbohydrate vs syngas fermentation. J. Chem. Technol. Biotechnol..

[7] Sánchez C (2009). Lignocellulosic residues: Biodegradation and bioconversion by fungi. Biotechnol. Adv..

[8] Bhardwaj N, Kumar B, Verma P (2019). A detailed overview of xylanases: An emerging biomolecule for current and future prospective. Bioresour. Bioprocess..

[9] Bornscheuer U, Buchholz K, Seibel J (2014). Enzymatic degradation of (ligno) cellulose. Angew. Chem. Int. Ed..

[10] Collins T, Gerday C, Feller G (2005). Xylanases, xylanase families and extremophilic xylanases. FEMS Microbiol. Rev..

[11] Peng F, Peng P, Xu F, Sun R-C (2012). Fractional purification and bioconversion of hemicelluloses. Biotechnol. Adv..

[12] Kim IJ, Jeong D, Kim SR (2022). Upstream processes of citrus fruit waste biorefinery for complete valorization. Bioresour. Technol..

[13] Paës G, Berrin J-G, Beaugrand J (2012). GH11 xylanases: Structure/function/properties relationships and applications. Biotechnol. Adv..

[14] Ko JK, Jung MW, Kim KH, Choi IG (2009). Optimal production of a novel endo-acting β-1,4-xylanase cloned from *Saccharophagus degradans* 2–40 into *Escherichia coli* BL21(DE3). N. Biotechnol..

[15] Nam KH, Park S, Park J (2022). Preliminary XFEL data from spontaneously grown endo-1,4-β-xylanase crystals from *Hypocrea virens*. Acta Crystallogr. F Struct. Biol. Commun..

[16] Lee J (1997). Biological conversion of lignocellulosic biomass to ethanol. J. Biotechnol..

[17] Chang T, Yao S (2011). Thermophilic, lignocellulolytic bacteria for ethanol production: Current state and perspectives. Appl. Microbiol. Biotechnol..

[18] Lee YE, Jain MK, Lee CY, Lowe SE, Zeikus JG (1993). Taxonomic distinction of saccharolytic thermophilic anaerobes: Description of *Thermoanaerobacterium*
*xylanolyticum* gen. nov., sp. nov., and *Thermoanaerobacterium*
*saccharolyticum* gen. nov., sp. nov.; Reclassification of *Thermoanaerobium*
*brockii*, *Clostridium*
*thermosulfurogenes*, and *Clostridium*
*thermohydrosulfuricum* E100-69 as *Thermoanaerobacter*
*brockii* comb. nov., *Thermoanaerobacterium*
*thermosulfurigenes* comb. nov., and *Thermoanaerobacter*
*thermohydrosulfuricus* comb. nov., respectively; and transfer of *Clostridium*
*thermohydrosulfuricum* 39E to *Thermoanaerobacter*
*ethanolicus*. Int. J. Bacteriol..

[19] Lin C-J (2010). Characterization of a thermophilic l-rhamnose isomerase from *Thermoanaerobacterium saccharolyticum* NTOU1. J. Agric. Food Chem..

[20] Andaleeb H (2020). High-resolution crystal structure and biochemical characterization of a GH11 endoxylanase from *Nectria haematococca*. Sci. Rep..

[21] Murshudov GN (2011). REFMAC5 for the refinement of macromolecular crystal structures. Acta Crystallogr. D.

[22] Williams CJ (2018). MolProbity: More and better reference data for improved all-atom structure validation. Protein Sci..

[23] Gouet P, Courcelle E, Stuart DI, Metoz F (1999). ESPript: Analysis of multiple sequence alignments in PostScript. Bioinformatics.

[24] Holm L, Rosenstrom P (2010). Dali server: Conservation mapping in 3D. Nucleic Acids Res..

[25] Paës G, Tran V, Takahashi M, Boukari I, O'Donohue MJ (2007). New insights into the role of the thumb-like loop in GH-11 xylanases. Protein Eng. Des. Sel..

[26] Wakarchuk WW, Campbell RL, Sung WL, Davoodi J, Yaguchi M (1994). Mutational and crystallographic analyses of the active site residues of the *Bacillus circulans* xylanase. Protein Sci..

[27] Hakulinen N, Turunen O, Janis J, Leisola M, Rouvinen J (2003). Three-dimensional structures of thermophilic β-1,4-xylanases from *Chaetomium*
*thermophilum* and *Nonomuraea*
*flexuosa*. Comparison of twelve xylanases in relation to their thermal stability. Eur. J. Biochem..

[28] Weinert T (2017). Serial millisecond crystallography for routine room-temperature structure determination at synchrotrons. Nat. Commun..

[29] Nam KH (2021). Room-temperature structure of xylitol-bound glucose isomerase by serial crystallography: Xylitol binding in the M1 site induces release of metal bound in the M2 site. Int. J. Mol. Sci..

[30] Knight JDR, Hamelberg D, McCammon JA, Kothary R (2009). The role of conserved water molecules in the catalytic domain of protein kinases. Proteins Struct. Funct. Genet..

[31] Herring CD (2016). Strain and bioprocess improvement of a thermophilic anaerobe for the production of ethanol from wood. Biotechnol. Biofuels.

[32] Hung K-S (2011). Characterization of a salt-tolerant xylanase from *Thermoanaerobacterium saccharolyticum* NTOU1. Biotechnol. Lett..

[33] Hung K-S (2011). Characterization of a novel GH10 thermostable, halophilic xylanase from the marine bacterium *Thermoanaerobacterium saccharolyticum* NTOU1. Process Biochem..

[34] Kim IJ, Bornscheuer UT, Nam KH (2022). Biochemical and structural analysis of a glucose-tolerant β-glucosidase from the hemicellulose-degrading *Thermoanaerobacterium saccharolyticum*. Molecules.

[35] Chakdar H (2016). Bacterial xylanases: Biology to biotechnology. 3 Biotech.

[36] Dheeran P, Nandhagopal N, Kumar S, Jaiswal YK, Adhikari DK (2012). A novel thermostable xylanase of *Paenibacillus macerans* IIPSP3 isolated from the termite gut. J. Ind. Microbiol. Biotechnol..

[37] York W, Oneill M (2008). Biochemical control of xylan biosynthesis—Which end is up?. Curr. Opin. Plant Biol..

[38] Ng CH, He J, Yang K-L (2015). Purification and characterization of a GH11 xylanase from biobutanol-producing *Clostridium beijerinckii* G117. Appl. Biochem. Biotechnol..

[39] Ghio S (2018). *Paenibacillus* sp. A59 GH10 and GH11 extracellular endoxylanases: Application in biomass bioconversion. Bioenergy Res..

[40] Bhat SK, Purushothaman K, Rao ARGRA, Kini RK (2022). Cloning and expression of a GH11 xylanase from *Bacillus pumilus* SSP-34 in *Pichia pastoris* GS115: Purification and characterization. J. Appl. Biol. Biotechnol..

[41] He J (2019). Design, expression and functional characterization of a thermostable xylanase from *Trichoderma reesei*. PLoS One.

[42] Mhlongo NN (2015). Dynamics of the thumb-finger regions in a GH11 xylanase *Bacillus circulans*: Comparison between the Michaelis and covalent intermediate. RSC Adv..

[43] Vucinic J (2021). A comparative study to decipher the structural and dynamics determinants underlying the activity and thermal stability of GH-11 xylanases. Int. J. Mol. Sci..

[44] Almagro Armenteros JJ (2019). SignalP 5.0 improves signal peptide predictions using deep neural networks. Nat. Biotechnol..

[45] Kim IJ, Youn HJ, Kim KH (2016). Synergism of an auxiliary activity 9 (AA9) from *Chaetomium globosum* with xylanase on the hydrolysis of xylan and lignocellulose. Process Biochem..

[46] Otwinowski Z, Minor W (1997). Processing of X-ray diffraction data collected in oscillation mode. Methods Enzymol..

[47] Park SY, Choi H, Eo C, Cho Y, Nam KH (2020). Fixed-target serial synchrotron crystallography using nylon mesh and enclosed film-based sample holder. Crystals.

[48] Lee D (2019). Nylon mesh-based sample holder for fixed-target serial femtosecond crystallography. Sci. Rep..

[49] Barty A (2014). Cheetah: Software for high-throughput reduction and analysis of serial femtosecond X-ray diffraction data. J. Appl. Crystallogr..

[50] White TA (2016). Recent developments in CrystFEL. J. Appl. Crystallogr..

[51] Gevorkov Y (2019). XGANDALF—Extended gradient descent algorithm for lattice finding. Acta Crystallogr. A Found. Adv..

[52] Vagin A, Teplyakov A (2010). Molecular replacement with MOLREP. Acta Crystallogr. D.

[53] Jumper J (2021). Highly accurate protein structure prediction with AlphaFold. Nature.

[54] Emsley P, Cowtan K (2004). Coot: Model-building tools for molecular graphics. Acta Crystallogr. D.

