# Supplementary material for: Characterization and structural analysis of the endo-1,4-β-xylanase GH11 from the hemicellulose-degrading Thermoanaerobacterium saccharolyticum useful for lignocellulose saccharification
Source: Sci Rep. 2023 Oct 13;13:17332. doi: 10.1038/s41598-023-44495-8 (PMC10576002; doi:10.1038/s41598-023-44495-8)
Supplement: Supplementary file 1 — Supplementary Figures. [file 41598_2023_44495_MOESM1_ESM.docx]

**Supplementary Information**

**Characterization and structural analysis of the *endo*-1,4-*β*-xylanase GH11 from the hemicellulose-degrading *Thermoanaerobacterium saccharolyticum* useful for lignocellulose saccharification**

In Jung Kim^1,2^, Soo Rin Kim^3^, Kyoung Heon Kim^4^, Uwe T. Bornscheuer^2^, Ki Hyun Nam^5,^*

^1^ Department of Food Science & Technology, Institute of Agriculture and Life Science, Gyeongsang National University, Jinju 52828, South Korea

^2^ Department of Biotechnology and Enzyme Catalysis, Institute of Biochemistry, University of Greifswald, Felix-Hausdorff-Str. 4, 17489 Greifswald, Germany

^3^ School of Food Science and Biotechnology, Kyungpook National University, Daegu, 41566, Korea

^4^ Department of Biotechnology, Graduate School, Korea University, Seoul, 02841, Korea

^5^ College of General Education, Kookmin University, Seoul, 02707, Korea

*Corresponding author: structure@kookmin.ac.kr


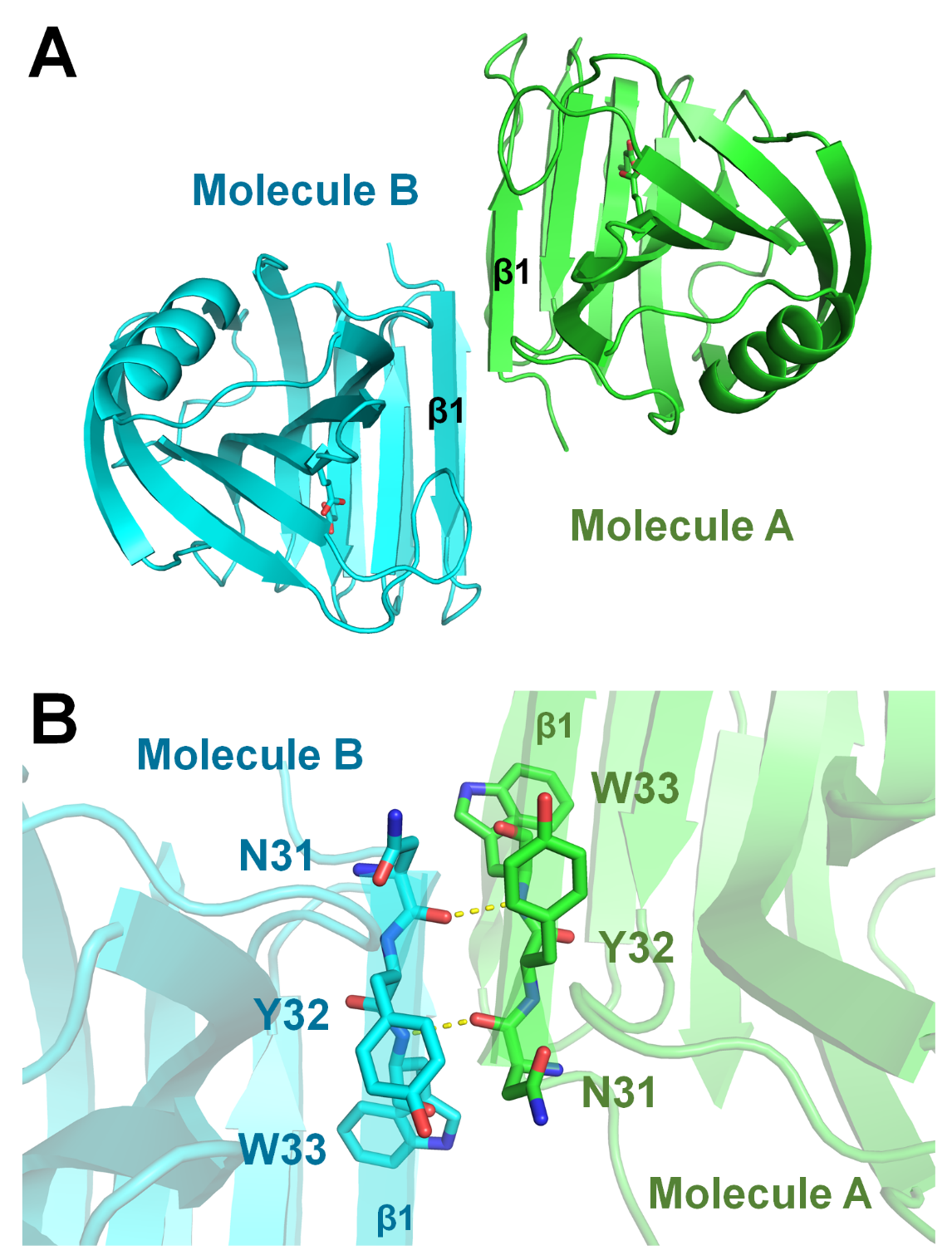


**Supplementary Figure S1.** (A) Two-fold symmetry crystal packing artifact dimer formation of TsaGH11 in the asymmetric unit. (B) Main chain interaction of β1-strands between two TsaGH11 molecules.


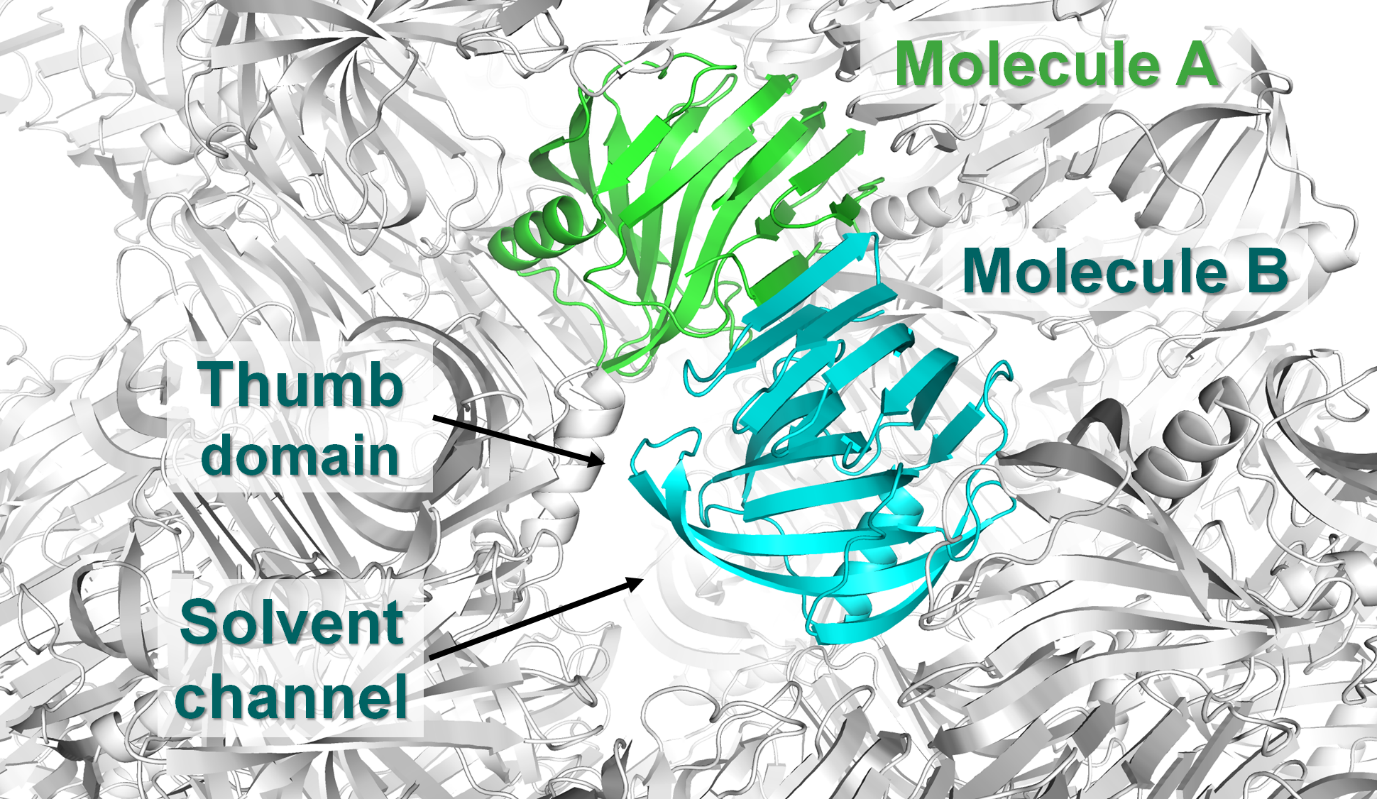


**Supplementary Figure S2**. Crystal packing of TsaGH11. The thumb and palm domain of TsaGH11B was exposed to a solvent channel in the crystal lattice.


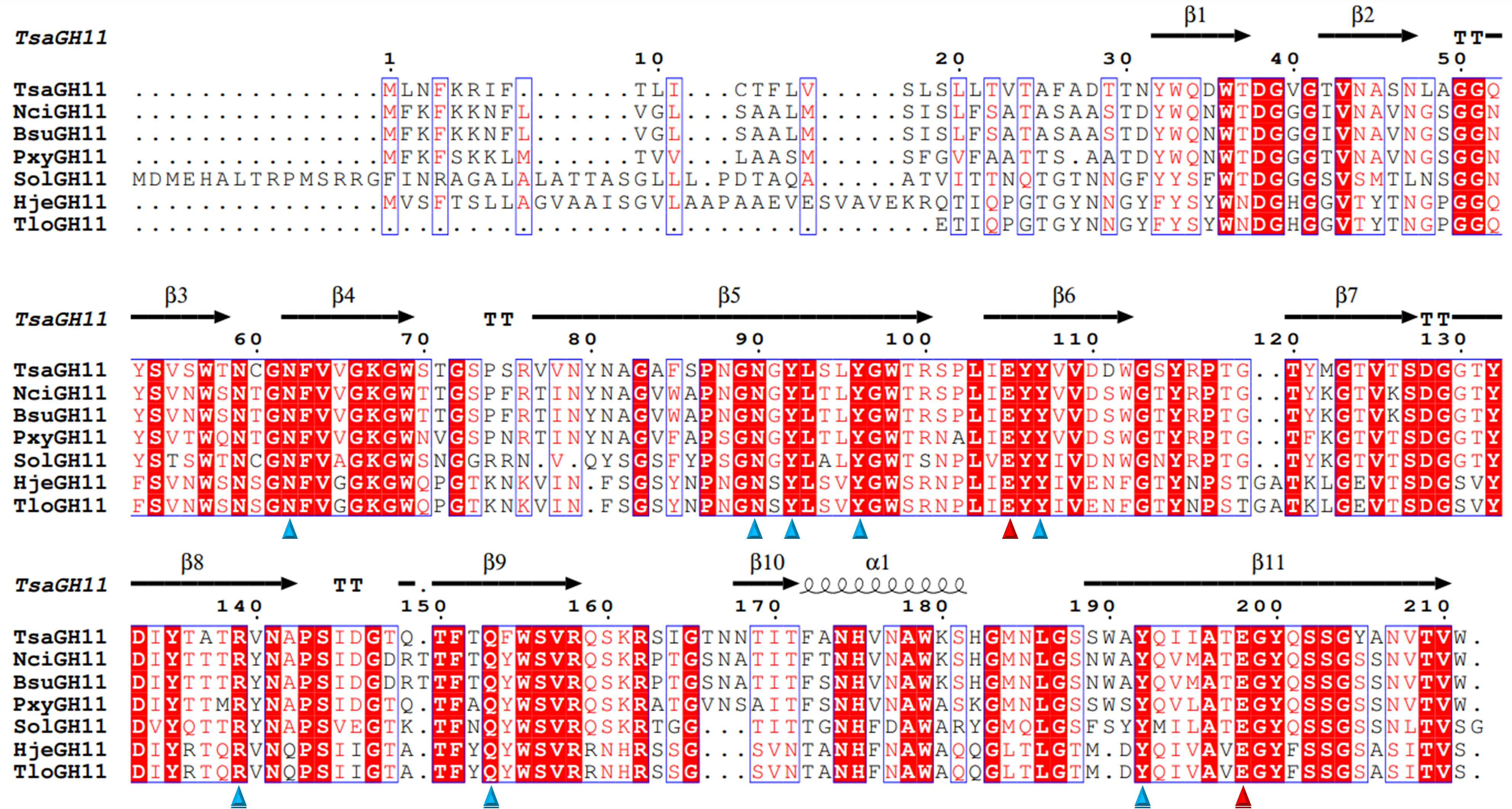


**Supplementary Figure S3**. Structure-based sequence alignment of TsaGH11 with GH11s from *Niallia circulans* (UniProt: P09850), *Bacillus subtilis* (P18429), *Paenibacillus xylanivorans* (A0A0M9BNX9), *Streptomyces olivaceoviridis* (A0A7G1MBT0), *Hypocrea jecorina* (P36217) and *Trichoderma longibrachiatum* (F8W669). The substrate binding and catalytic residues are indicated by blue and red triangles, respectively.


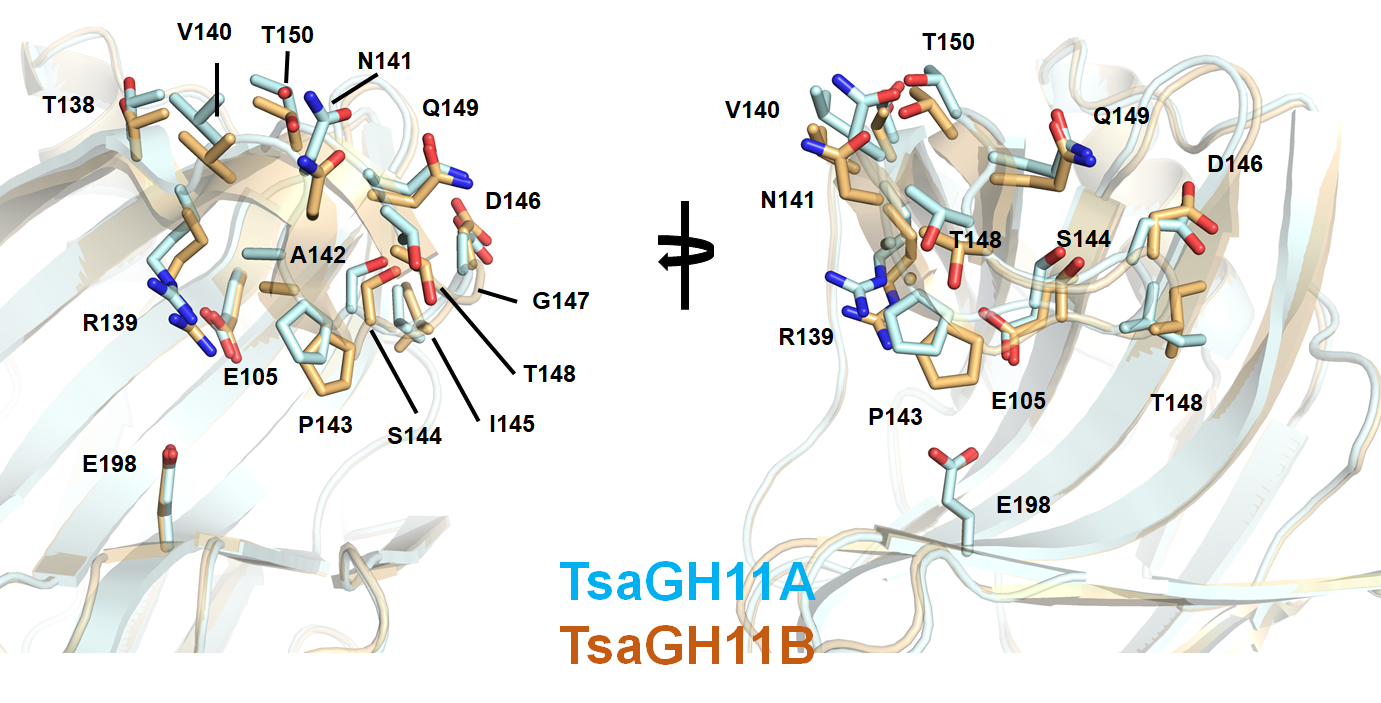


**Supplementary Figure S4**. Close-up view of the difference in conformation of the thumb domain from superimposition of TsaGH11A and TsaGH11B molecules.


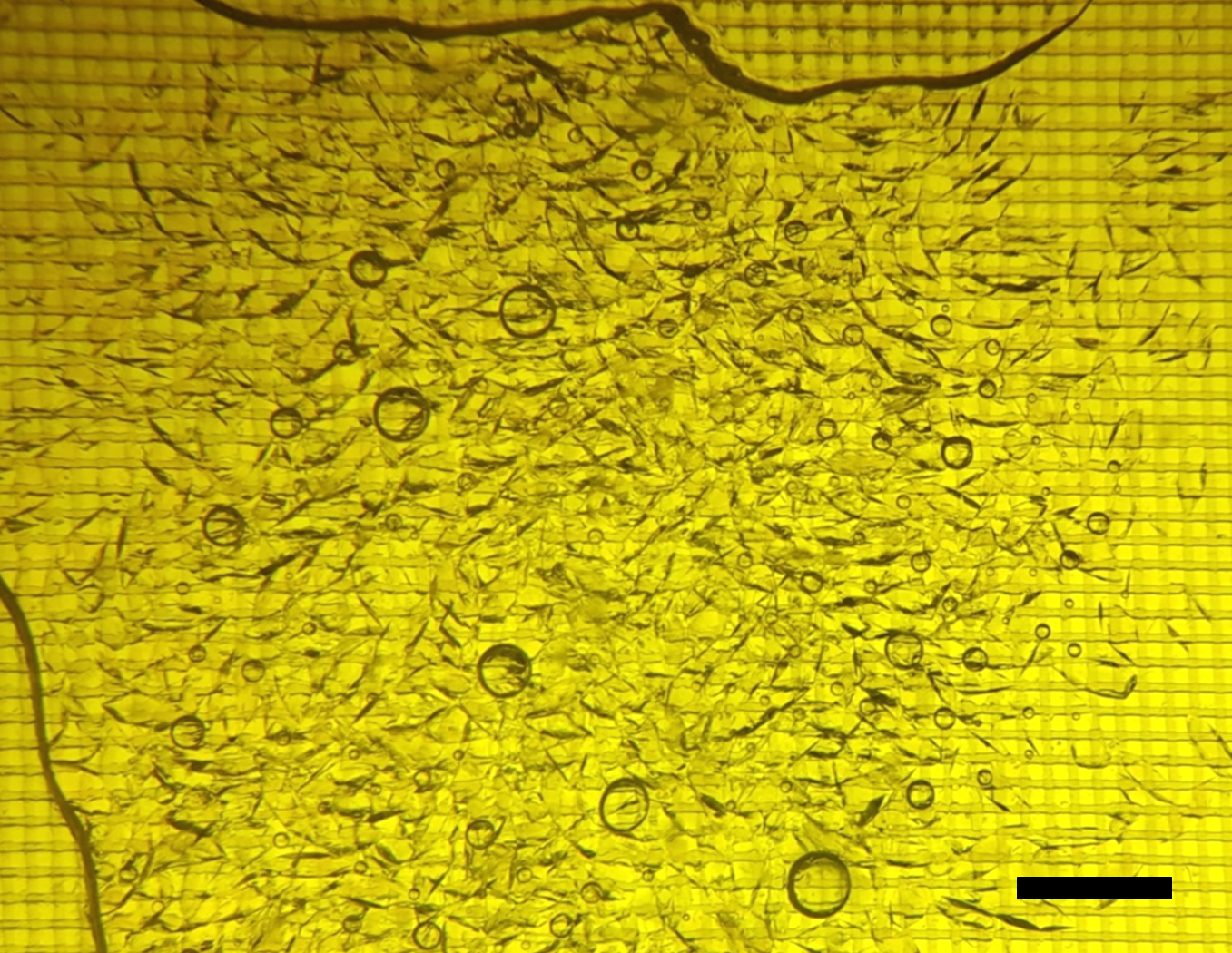


**Supplementary Figure S5**. Photo of TsaGH11 crystals on nylon mesh and enclosed film (NAM)-based sample holder. The scale bar indicates 600 μm.


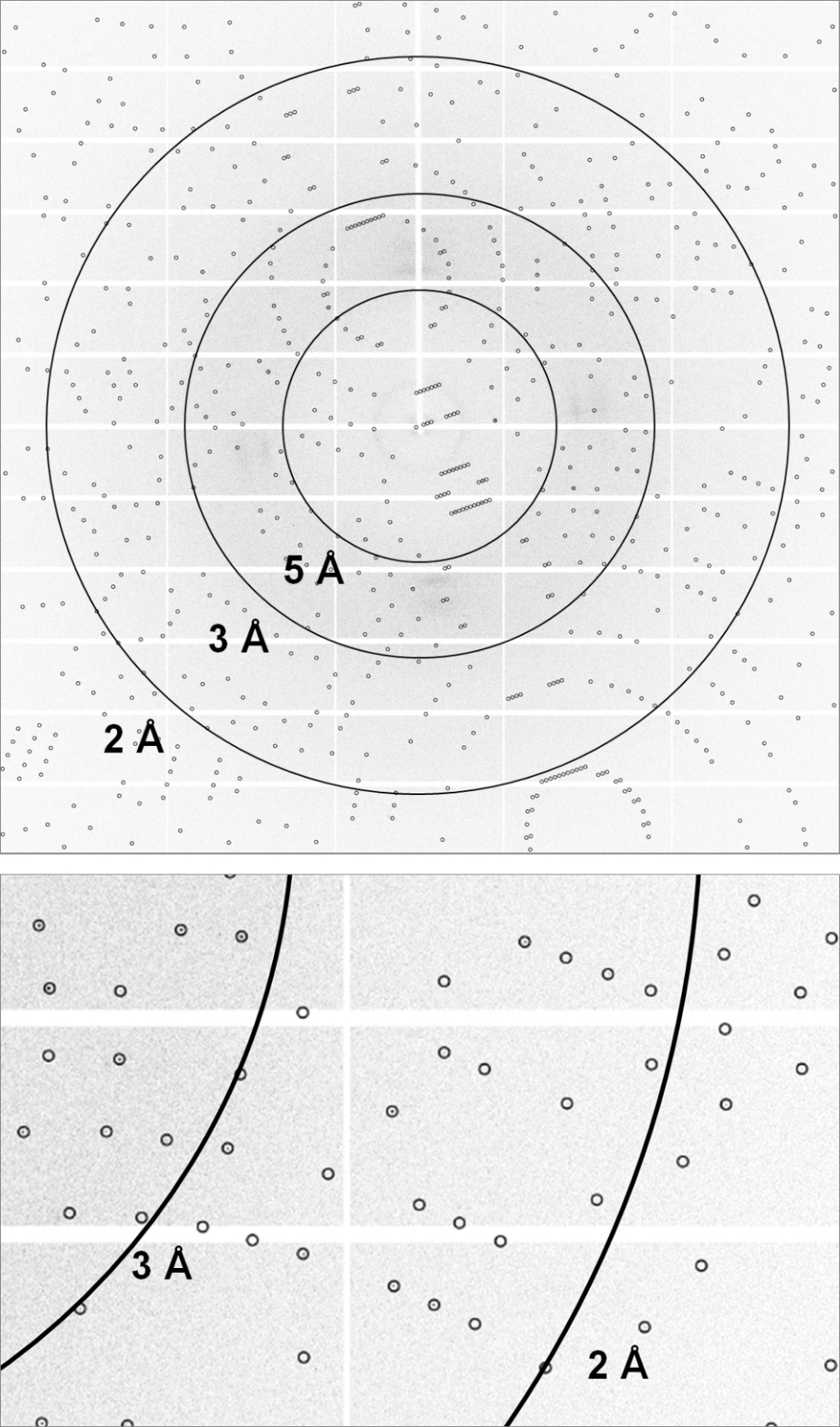


**Supplementary Figure S6**. Typical diffraction patterns of TsaGH11 delivered to NAM-based sample holder. The circles indicate the predicted locations of Bragg peaks. (Bottom) close-up view of indexed Bragg peaks.


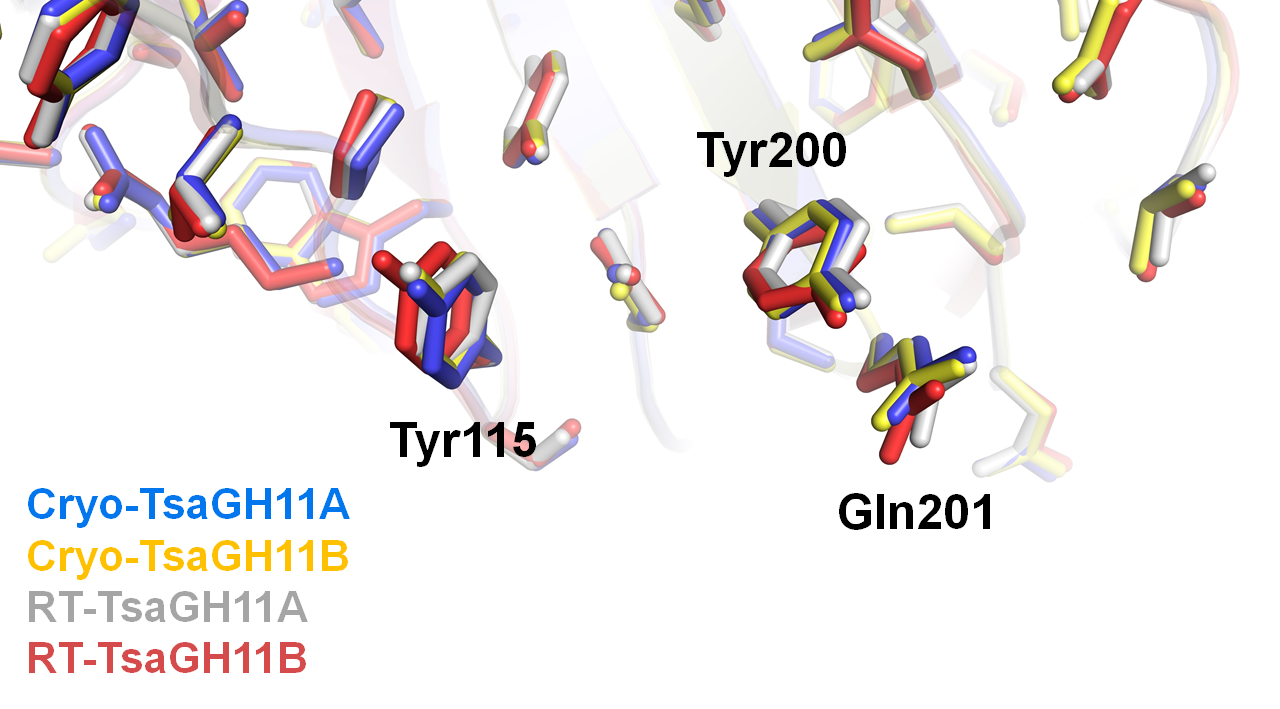


**Supplementary Figure S7.** Superimposition of room-temperature and cryogenic structures of TsaGH11. Close-up view of the positional difference of the side chain of Tyr115, Tyr200 and Gln201 in thumb domain. Tyr115 and Tyr200 are involved in the substrate binding.


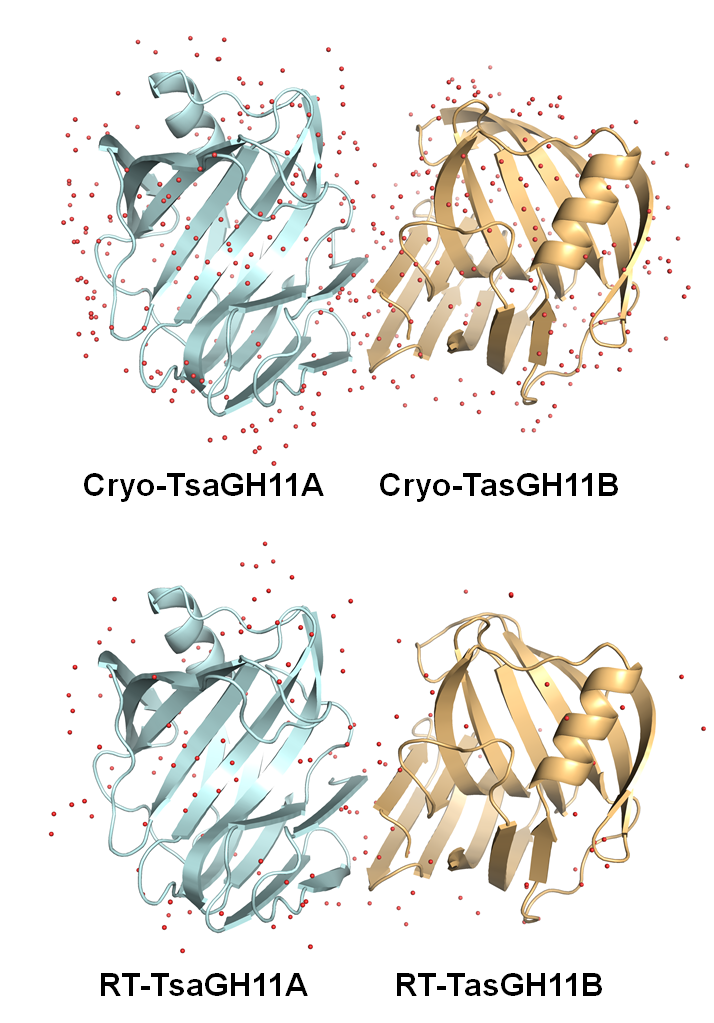


**Supplementary Figure S8.** Distribution of water molecules (red sphere) in Cryo-TsaGH11 and RT-TsaGH11 crystal structures.
